# Directed evolution and modular integration of a high-affinity ICOS-L variant for potent T cell–mediated tumor elimination

**DOI:** 10.1186/s13036-025-00536-6

**Published:** 2025-07-11

**Authors:** Ji Yeon Ha, Tae Wook Song, Petrina Jebamani, Sun-Gu Lee, Sang Taek Jung

**Affiliations:** 1 Department of Biomedical Sciences, Graduate School, Korea University, Seoul, 02841 Republic of Korea; 2 BK21 Graduate Program, Department of Biomedical Sciences, Korea University College of Medicine, Seoul, 02841 Republic of Korea; 3 Department of Chemical and Biological Engineering, Seoul National University, Seoul, 08826 Republic of Korea; 4 Department of Chemical Engineering, Pusan National University, Busan, 46241 Republic of Korea; 5 Interdisciplinary Program for Bioengineering, Seoul National University, Seoul, 08826 Republic of Korea; 6 Institute of Chemical Processes, Seoul National University, Seoul, 08826 Republic of Korea; 7 BioMAX, Seoul National University, Seoul, 08826 Republic of Korea; 8 Seoul National University Medical Research Center (SNUMRC), Seoul, 03080 Republic of Korea

**Keywords:** ICOS-L, T-cell co-stimulation, Immune checkpoint fusion, Directed evolution, Synthetic Immunomodulator, Modular biologics

## Abstract

**Background:**

Advancing cancer immunotherapy requires engineering synthetic immunomodulators that integrate precise receptor targeting, tunable activity, and compatibility with modular biologic formats. The Inducible T-cell Co-Stimulator (ICOS) is a clinically validated co-stimulatory receptor whose engagement enhances T-cell function. However, the development of ICOS-targeting biologics has been hindered by limited receptor affinity and format-dependent agonist activity. To address this, we applied a protein engineering framework to optimize the ICOS ligand (ICOS-L) as a high-affinity, modular component for precision immune modulation.

**Results:**

Using yeast surface display–based directed evolution, we identified an ICOS-L variant (Y8) containing two synergistic mutations (Q51P and N57H) that improved human ICOS (hICOS) binding affinity by ~ 100-fold relative to wild-type. Structural modeling revealed that Q51P enhances backbone rigidity via a proline-induced conformational constraint, while N57H introduces a salt bridge with Asp86 in hICOS. These mutations reconfigure the receptor-binding interface to support high-affinity engagement. Functionally, Y8 induced potent T-cell proliferation and IFN-γ secretion. When genetically fused to pembrolizumab, Y8 further enhanced T-cell activation and tumor cell lysis, demonstrating synthetic synergy between PD-1 blockade and ICOS agonism. Among fusion formats, light-chain conjugation (pembrolizumab-L-Y8) exhibited superior functional output, highlighting the importance of geometric configuration in optimizing fusion-based agonism.

**Conclusion:**

This study establishes Y8 as a high-affinity ICOS-L variant with robust co-stimulatory function, capable of potentiating anti–PD-1 immunotherapy through modular fusion design. The integration of Y8 into therapeutic antibody scaffolds provides a versatile engineering framework for the development of next-generation immunomodulatory biologics, offering opportunities to overcome resistance and enhance clinical efficacy in cancer immunotherapy.

**Supplementary Information:**

The online version contains supplementary material available at 10.1186/s13036-025-00536-6.

## Introduction

Recent advances in synthetic biology and therapeutic protein engineering have enabled the rational modulation of immune signaling pathways through the design of structurally optimized ligands, receptors, and antibody fusion formats [1, 2]. In particular, engineering co-stimulatory immune checkpoint modulators with improved receptor targeting and programmable architecture represents a powerful strategy to augment T cell activation while overcoming the limitations of current immune checkpoint inhibitors (ICIs) [3–5]. Such approaches are expanding the immunotherapy toolbox beyond conventional antibody blockade, enabling the development of next-generation immunotherapeutics that integrate precision, potency, and modularity [6, 7].

The development of immune checkpoint inhibitors (ICIs) has transformed the landscape of cancer therapy by targeting key inhibitory pathways that limit antitumor immune responses. ICIs primarily function by blocking inhibitory immune checkpoints such as Programmed Death-1 (PD-1) and its ligand PD-L1, which deliver negative signals that suppress T-cell activity [8]. Despite the remarkable clinical success of ICIs, many patients do not respond to them or develop resistance over time. Despite their status as a cornerstone of modern immunotherapy, PD-1 inhibitors elicit heterogeneous and frequently suboptimal responses. Resistance arises from T cell exhaustion, compensatory checkpoint upregulation, and insufficient co-stimulatory signaling within the tumor microenvironment. The response rates for these therapies vary by cancer type, and many patients eventually develop resistance, highlighting the need for improved therapeutic strategies [9, 10]. A major factor underlying resistance is the elevated expression of various immune checkpoints, which makes it challenging to block all inhibitory signals simultaneously [11, 12]. To address this challenge, engineering agonists targeting co-stimulatory receptors has emerged as a complementary strategy to restore and potentiate T cell function. Such strategies are designed not only to relieve PD-1–mediated suppression but also to reinvigorate effective immune activation, particularly in tumors unresponsive to checkpoint monotherapy. These co-stimulatory molecules provide positive signals to T cells, promoting their proliferation, survival, and differentiation, thereby boosting immune responses against cancer. Integrating engineering principles such as modular fusion design, structure-guided mutagenesis, and directed evolution enables the creation of immune modulators with tunable activity and enhanced therapeutic performance [4–6, 13, 14].

Among co-stimulatory receptors, ICOS (Inducible T-cell Co-Stimulator) plays a central role in sustaining T cell responses during later stages of activation. ICOS interacts with its ligand, ICOS-L (B7-H2, CD275), which is expressed on various cells, including tumor cells and antigen-presenting cells in the tumor microenvironment [15]. Activation of ICOS signaling triggers the PI3K and AKT pathways and plays a multifaceted role in sustaining effector T-cell responses [16], driving memory T-cell formation [17], and supporting T cell–dependent B-cell immunity [18], which are important for humoral immune reactions. ICOS agonists have significant potential as therapeutic agents [19, 20]. Prior studies have highlighted the importance of ICOS in enhancing combination therapy with anti-CTLA-4 treatments [21], as seen in bladder cancer patients in whom ICOS-expressing T cells increased after therapy. ICOS agonists, such as the anti-ICOS IgG4 monoclonal antibody feladilimab, have shown promising efficacy and low toxicity in patients with advanced solid tumors when combined with anti-PD-1 therapies. Feladilimab, which induces but does not deplete ICOS^+^ T cells, has demonstrated antitumor activity across nonclinical and clinical models, and preliminary clinical trials suggest its potential benefit when combined with PD-1 inhibitors in patients with solid tumors [22].

Although antibody-based ICOS agonists have shown clinical promise, their functional activity is often difficult to predict and frequently requires iterative optimization of binding interfaces, valency, and molecular format. These challenges arise from the inherent uncertainty in whether a given antibody will act as an agonist, antagonist, or inert binder, necessitating labor-intensive functional screening. In contrast, ligand-based strategies offer a rational alternative by leveraging evolutionarily conserved receptor-binding interfaces that naturally support productive signaling. ICOS-L, the physiological ligand of ICOS, serves as a structurally compatible and rationally engineerable scaffold that engages the receptor through biologically optimized geometry and orientation [23]. This compatibility enables predictable receptor activation and facilitates modular incorporation into formats such as Fc- and antibody-fusions. In addition, its compact and genetically tractable nature allows for flexible reconfiguration in engineered constructs, supporting programmable agonistic functions. From an engineering perspective, this approach offers a modular and rational framework for designing ligand-based immune agonists with minimized dependence on empirical trial-and-error.

Despite these advantages, the wild-type ICOS-L exhibits relatively weak binding affinity to hICOS, with a dissociation constant (K_D_) in the hundreds of nanomolar range, necessitating a marked improvement in this binding affinity to make the ICOS-L ectodomain a more effective hICOS agonist. A prior study by Levin et al. (Frontiers in Immunology, 2020) described ICOS-L variants with improved binding to hICOS and demonstrated that their functional activity was highly dependent on molecular presentation—soluble forms exhibited antagonist-like behavior, while cross-linked variants induced agonism [24]. However, the affinity gains were modest, and no clear relationship between binding strength and functional potency was established. In this study, we implemented an integrated protein engineering strategy that combines directed evolution, structural modeling, and therapeutic fusion design to generate ICOS-L variants with ~ 100-fold higher affinity than wild-type. Functional characterization across multiple cellular assays consistently revealed that enhanced receptor binding translated into stronger agonistic responses. This framework not only supports a mechanistic understanding of the affinity–function relationship but also offers a generalizable strategy for optimizing ligand-based immune agonists in therapeutically relevant designs.

In this study, we applied a directed evolution strategy to engineer the ICOS-L ectodomain for enhanced affinity to hICOS, using yeast surface display to isolate variants with markedly improved binding, followed by structural modeling to gain mechanistic insight into enhanced receptor engagement. This approach yielded the Y8 variant, incorporating two key mutations (Q51P and N57H) that together increased ICOS affinity by nearly 100-fold compared to wild-type ICOS-L, primarily through reduced dissociation rates. To demonstrate translational potential, we fused the engineered ICOS-L variants to pembrolizumab (anti–PD-1), generating dual-function biologics capable of simultaneous checkpoint blockade and T cell co-stimulation. These ICOS-L fusion constructs exemplify an engineering-driven platform for constructing synthetic immunomodulators with enhanced bioactivity, tumor penetration, and modular therapeutic applications. The integration of affinity maturation, structural analysis, and modular antibody fusion design highlights the utility of protein engineering frameworks. These strategies collectively enable the development of next-generation immune checkpoint therapeutics with enhanced translational potential.

## Materials and methods

### Reagents

All oligonucleotide primers listed in Table S1 were synthesized by Cosmogenetech (Seoul, Korea) and Bionics (Seoul, Korea). Vent polymerase, restriction endonucleases, and the Gibson Assembly Master Mix were purchased from New England BioLabs (Ipswich, MA, USA). Yeast nitrogen base without amino acids, casamino acids, peptone, and yeast extract were obtained from Becton Dickinson Diagnostic Systems (Sparks, MD, USA). Disodium hydrogen phosphate anhydrous (Na_2_HPO_4_) and calcium chloride dihydrate (CaCl_2_∙2H_2_O) were purchased from Junsei (Tokyo, Japan). D-(+)-galactose, lithium acetate dihydrate (LiAc), D-sorbitol, L-tryptophan, histopaque-1077, and human AB serum were purchased from Sigma-Aldrich (St. Louis, MO, USA). Sodium phosphate monobasic monohydrate (NaH_2_PO_4_), glucose, 1,4-dithiolthreitol (DTT), and polyethylenimine (PEI)-Max were obtained from Samchun Chemical (Seoul, Korea), Duksan (Seoul, Korea), Roche (Basel, Switzerland), and Polysciences (Taipei, Taiwan), respectively. QuikChange Site-Directed Mutagenesis Kit and Frozen-EZ Yeast Transformation II Kit were supplied by Agilent Technologies (Santa Clara, CA, USA) and Zymo Research (Irvine, CA, USA), respectively. CD14^+^ MicroBeads, CD4^+^ T Cell Isolation Kit, and Pan T Cell Isolation Kit were acquired from Miltenyi Biotec (Bergisch Gladbach, Germany). Ham’s F12 medium, RPMI 1640 medium, trypsin-EDTA, fetal bovine serum (FBS), antibiotic-antimycotic, sodium pyruvate, MEM non-essential amino acids solution, FreeStyle 293 expression medium, Alexa Fluor 488 Protein Labeling Kit, and CFSE Cell Proliferation Kit were supplied by Thermo Fisher Scientific (Waltham, MA, USA). Interleukin-4 (IL-4), tumor necrosis factor-α (TNF-α), interleukin-6 (IL-6), interleukin-1β (IL-1β), and Human Cytokine DuoSet ELISA Kit were purchased from R&D Systems (Minneapolis, MN, USA). Interleukin-2 (IL-2) and granulocyte-macrophage colony-stimulating factor (GM-CSF) were obtained from Peprotech (Cranbury, NJ, USA). Prostaglandin E_2_ (PGE_2_) was supplied by Tocris Bioscience (Bristol, England). Ni-NTA agarose, Protein A resin, and Glutathione resin were purchased from Qiagen (Hilden, Germany), Puriogen (Yeosu, Korea), and GenScript (Piscataway, NJ, USA), respectively.

### Construction of plasmids

All plasmids used in this study are listed in Table S2. cDNAs encoding wild-type ICOS-L (D1-T238), the ICOS-L variant A184 [24], hICOS (E21-K140), and mouse ICOS (E21-K141)-hFc were synthesized by Twist Bioscience (San Francisco, CA, USA). DNA fragments encoding the hICOS ectodomain were PCR amplified using the following primer sets: JYI#1/JYI#21 for hICOS-His, JYI#1/JYI#3 for hICOS-GST, and JYI#1/JYI#4 for hICOS-hFc. GST and Fc fragments were amplified using primers HW#7/JYI#81 and JYI#5/JYP#192. These fragments were assembled via primers JYI#1/JYI#81 and subcloned into the pMAZ vector using *Bss*HII/*Xba*I digestion, generating pMAZ-hICOS-His, pMAZ-hICOS-GST and pMAZ-hICOS-Fc plasmids.

For yeast surface display, ICOS-L wild-type and A184 were amplified using primers JYI#8/JYI#9 and cloned into pCTCON vectors using *Sfi*I sites, generating pCTCON-Aga2-ICOS-L constructs. For mammalian expression, ICOS-L variants (wild-type, A184, and Y8) were PCR amplified with primers JYI#6/JYI#7 for wild-type and A184, and JYI#14/JYI#15 for Y8. These were ligated into pMAZ vectors using *Bss*HII/*Xba*I sites to generate pMAZ-ICOS-L-His plasmids. The Y8 variant was assembled using Gibson Assembly, and single mutations were introduced using the QuikChange Site-Directed Mutagenesis Kit with primers (JYI#16/JYI#17 for N57H, JYI#18/JYI#19 for Q51P).

To construct ICOS-L variants fused with IgG1 Fc (LALAPG; effector function-silencing mutations from Roche), ICOS-L variants and Fc (LALAPG) were PCR amplified using primers JYI#14/JYI#23 and JYI#24/JYI#25, then assembled using JYI#14/JYI#40. For IgG4 Fc fusion with in-house Fc mutations for silent effector function (Fc-S) and extended half-life (Fc-H), pembrolizumab heavy chain genes were amplified using primers JYI#46/JYI#47 and JYI#48/JYI#49, assembled with JYI#46/JYI#50, and cloned into pMAZ vectors to generate pMAZ-pembrolizumab-IgH (Fc-SH). To generate ICOS-L variants fused with IgG4 Fc (Fc-SH), the ICOS-L and Fc-SH fragments were amplified using primers JYI#77/JYI#78 and JYI#79/JYI#80 and assembled with JY#33/JY#34. The resulting mammalian expression vectors for ICOS-L-Fc (IgG4 Fc-SH), including pMAZ-ICOS-L-wild-type-Fc and pMAZ-ICOS-L-Y8-Fc, were constructed via Gibson Assembly.

For pembrolizumab-ICOS-L constructs, heavy and light chains of pembrolizumab (IgH and IgL) were PCR amplified using primers JYI#46/JYI#55 and JYI#60/JYI#61, respectively. ICOS-L variants were amplified using primers JYI#56/JYI#57 and assembled with pembrolizumab IgH and IgL using primers JYI#58/JYI#59 and JYI#62/JYI#59, generating pMAZ-pembrolizumab-IgH-ICOS-L and pMAZ-pembrolizumab-IgL-ICOS-L constructs.

### Mammalian cell expression and purification of ICOS, ICOS-L variants, and ICOS-L fusion proteins

ICOS-L variant expression plasmids (e.g., pMAZ-ICOS-L-wild-type-His, pMAZ-ICOS-L-A184-His, pMAZ-ICOS-L-Y8(Q51P, N57H)-His, pMAZ-ICOS-L-H(N57H)-His, pMAZ-ICOS-L-P (Q51P)-His, and ICOS expression plasmids (e.g., pMAZ-hICOS-His, pMAZ-hICOS-GST, pMAZ-hICOS-Fc, and pMAZ-mICOS-Fc), and plasmids for ICOS-L variants fused to IgG1 Fc (LALAPG), IgG4 Fc with silent effector and extended half-life mutations, and pembrolizumab were transfected into Expi293F cells using PEI-Max for transient expression. After seven days of culture, supernatants were collected and processed using the appropriate affinity resin (Ni-NTA agarose, glutathione resin, or Protein A resin). After extensive washing of the column, elution was performed using 1× PBS containing 250 mM imidazole (pH 7.4), 50 mM Tris-HCl containing 10 mM GSH (pH 8.0), or 100 mM glycine-HCl (pH 2.7), followed by buffer exchange into 1× PBS (pH 7.4) using Amicon Ultra-4 Filters (Merck Millipore, Burlington, MA, USA), and protein concentration was measured using an Epoch plate reader equipped with a Take3 Plate (BioTek, Winooski, VT, USA). For additional purification, size exclusion chromatography (SEC) was conducted. 1 mL of purified proteins was loaded onto a Superdex^®^ Increase 200 column (Cytiva) and eluted with 1× PBS at 0.7 mL/min using NGC chromatography system (Bio-Rad, Hercules, CA, USA).

### Library construction and flow cytometric screening

The gene encoding ICOS-L wild-type was randomly mutated using primers JY#28/JY#29 via error-prone PCR and amplified with primers JY#30/JY#31. The amplified genes and the pCTCON-Aga2-FLAG plasmid were digested with *Sfi*I and transformed into the AWY101 strain by homologous recombination to construct the library. The library was cultured in 500 mL of SDCAA (20 g/L glucose, 6.7 g/L yeast nitrogen base without amino acids, 5 g/L casamino acids, 5.4 g/L Na_2_HPO_4_, 8.56 g/L NaH_2_PO_4_) supplemented with 50 µg/mL of kanamycin and 40 µg/mL of chloramphenicol at 30 °C for 16 h. Cells were then adjusted to OD_600_ = 0.7 in 100 mL fresh SDCAA and incubated for 16 h at 30 °C. Next, they were transferred to 100 mL of SGCAA (20 g/L galactose, 6.7 g/L yeast nitrogen base without amino acids, 5 g/L casamino acids, 5.4 g/L Na_2_HPO_4_, 8.56 g/L NaH_2_PO_4_) (OD_600_ = 0.7) and incubated at 20 °C for 2 days. After induction, 1 × 10^8^ cells were harvested, washed with 1 mL of PBSB (0.1% BSA in PBS), centrifuged at 14,000 × g, and labeled with anti-FLAG-iFluor647 (1,000:1) and hICOS-Fc-Alexa488 probes for 1 h at room temperature. The cells were washed twice with PBSB, and those displaying ICOS-L variants with the top 0.2–0.3% fluorescence intensity were gated and sorted using an S3 cell sorter (Bio-Rad, Hercules, CA, USA). The recovered yeasts were cultured in 20 mL of SDCAA at 30 °C, harvested the next day, and transferred to 100 mL of SGCAA for incubation at 20 °C for 2–3 days. This screening process was repeated four times with decreasing probe concentrations.

### Enzyme-linked immunosorbent assay (ELISA)

To analyze ICOS or PD-1 binding, 50 µL of hICOS-Fc, hICOS-Fc, mICOS-Fc, hICOS-GST, and PD-1-GST (4 µg/mL in 0.05 M Na_2_CO_3_, pH 9.6) were coated onto 96-well flat-bottom polystyrene high-binding microplates (Costar, Corning, NY, USA) and incubated 4 °C overnight. For simultaneous binding analysis of pembrolizumab fused with an ICOS-L variant to ICOS and PD-1, 48 nM of PD-1-Fc was coated and incubated under the same conditions. All plates were blocked with 0.5% BSA in PBS (pH 7.4) for 1 h, then washed four times with PBST (PBS containing 0.05% Tween20, pH 7.4). For ICOS binding, 50 µL of serially diluted ICOS-L variants in blocking solution was added, incubated for 1 h, and washed four times in PBST. Next, 50 µL of mouse anti-His–HRP conjugate was added, incubated for 1 h, and washed again. For ICOS-L fusion protein binding to ICOS or PD-1, serially diluted fusion proteins in 1% skim milk were added, followed by the addition of Protein L or Protein A-HRP conjugate. For dual-target binding, 240 nM pembrolizumab-ICOS-L variants were added to PD-1-Fc coated plates, followed by serially diluted monomeric ICOS proteins and subsequently mouse anti-His-HRP conjugate. After incubation, all plates were washed, developed with the 1-Step Ultra TMB ELISA Substrate (Thermo Fisher Scientific, Waltham, MA, USA), quenched with 4 *N* H_2_SO_4_, and absorbance was measured at 450 nm using an Epoch plate reader (BioTek, Winooski, VT, USA).

### Bio-layer interferometry (BLI) assay

The affinity constants of ICOS-L variants (wild-type, A184, Y8, Q51P, and N57H) for ICOS were analyzed using a bio-layer interferometry assay with the Octet R8 (Satorius, Göttingen, Germany). hICOS-Fc (5 µg/mL) was immobilized on a Protein A biosensor (Satorius, Göttingen, Germany) equilibrated with 1× PBS (pH 7.4) for 1 min, followed by a baselining with 1× PBS (pH 7.4) for 1 min. Next, serial dilutions of ICOS-L variants were added for a 10 min association phase, followed by a 10 min dissociation phase in 1× PBS (pH 7.4). The BLI signals were recorded and analyzed using Octet Analysis Studio software (Satorius, Göttingen, Germany).

### Modeling of ICOS-ICOS-L variant complex structure and identification of interactions

The wild-type structure of the hICOS–ICOS-L complex (PDB ID: 6X4G) [23] was obtained from the Protein Data Bank (PDB) (https://www.rcsb.org/) [25]. For this study, only chain A (hICOS) and chain C (ICOS-L) were retained for analysis, with all other chains and ligands excluded to focus on the hICOS-ICOS-L interaction. Using PYMOL tool [26], the wild-type ICOS-L was mutated to generate the Y8 variant, incorporating the Q51P and N57H mutations. The modified structure was evaluated for any missing atoms or imperfections using SPDBV tool (http://www.expasy.org/spdbv/) [27]. Energy minimization was then performed using GROMACS (GROningen MAchine for Chemical Simulation) [28, 29] with the CHARMM27 [30] force field applied as follows. The protein was placed in a cubic unit cell, maintaining a 1.0 nm distance from the edge, and solvated with the SPC/E water model. Na^+^ or Cl^–^ ions were added as needed to neutralize the system’s charge, followed by energy minimization via the steepest descent algorithm for over 50,000 steps to achieve structural stability. Non-covalent interactions between the mutated ICOS-L variant and ICOS were identified using PPCheck. (https://caps.ncbs.res.in/ppcheck/index.html) [31].

### Isolation of peripheral blood mononuclear cells (PBMCs)

PBMCs were isolated from fresh blood of a healthy donor by density gradient centrifugation using Histopaque-1077. After centrifugation at room temperature for 10 min (1000 × g, brake off), PBMCs were collected, transferred to a new tube, and washed twice with 1× MACS buffer (2 mM EDTA, 0.5% BSA in PBS), followed by centrifugation at 100 × g for 10 min.

### Cell binding analysis

T cells were isolated from freshly prepared PBMCs using a pan T cell isolation kit. The isolated T cells were then cultured and activated in complete RPMI 1640 medium supplemented with IL-2 and Human CD3/CD28 T Cell Activator (Stemcell Technologies, Vancouver, Canada) for 3 days. After activation, T cells were harvested and treated with ICOS-L-Fc (LALAPG). Cells were then labeled with Protein A-FITC for 30 min on ice. PD-1-expressing CHO cells, prepared in-house using the Flp-In system (Thermo Fisher Scientific, Waltham, MA, USA), were cultured in F12 medium supplemented with 10% FBS and harvested. 5 × 10^6^ cells were treated with ICOS-L fusion proteins, then labeled with Protein A-FITC and ICOS-Fc-Alexa488 for 30 min on ice. After washing with 1× EDTA buffer (Bio-Rad, Hercules, CA, USA), the cells were analyzed using a FACSLyric instrument (BD Biosciences, Franklin Lakes, NJ, USA).

### T cell activation assay

Freshly isolated T cells were stained with carboxyfluorescein diacetate succinimidyl ester (CFSE) and seeded at 5 × 10^4^ cells/well in flat-bottom 96 well plates coated with anti-CD3 (OKT3, R&D Systems, Minneapolis, MN) and sample proteins. After 3.5 days of cultivation, cells were harvested and analyzed for T cell proliferation using a FACSLyric instrument. The supernatants were analyzed for cytokine secretion using the Human IFN-gamma DuoSet ELISA kit.

### Mixed lymphocyte reaction (MLR) assay

CD14^+^ monocytes were isolated from PBMCs of healthy donor 1 using MACS technology using CD14 MicroBeads. To generate immature dendritic cells (iDCs), isolated monocytes (3 × 10^6^ cells/well) were cultured in 3 mL of complete RPMI medium (RPMI 1640, 10% FBS, antibiotic-antimycotic) supplemented with 50 ng/mL of IL-4 and 50 ng/mL of GM-CSF and incubated at 37 °C with 5% CO_2_ for 6 days. iDCs were then matured by culturing in complete medium supplemented with 20 ng/mL of TNF-α, 20 ng/mL of IL-6, 10 ng/mL of IL-1β, and 1 µg/mL of PGE2 for 24 h to generate mature DCs (mDCs). CD4^+^ T cells were isolated from PBMCs of healthy donor 2 using MACS technology and a CD4^+^ T cell isolation kit. The T cells were stained with CFSE according to the manufacturer’s instructions. Mature DCs (1 × 10^4^) and allogenic CD4^+^ T cells (1 × 10^5^) were cocultured with different concentrations of protein samples in MLR medium (RPMI 1640, 2 mM L-glutamine, nonessential amino acids, 0.1 mM sodium pyruvate, 5% human AB serum). After 5 days of co-culture, cells were harvested, and the supernatants were collected. T cell proliferation was determined by measuring CFSE fluorescence using a FACSLyric instrument. The levels of secreted IFN-ɣ and IL-2 in the supernatants were measured by ELISA according to the manufacturer’s instructions.

### T cell-mediated tumor lysis assay

MDA-MB-231 and A375 tumor cells were cultured at a density of 10,000 cells per well in an E-Plate. In parallel, T cells were activated for 3 days using a Human CD3/CD28 T Cell Activator. One day after seeding the tumor cells, the activated T cells were counted, and 5,000 cells per well were co-cultured with the attached tumor cells. Anti-CD3 antibody and ICOS-L fusion proteins were simultaneously added to the wells. T cell-mediated tumor cell lysis was assessed by monitoring the cell index and % cytolysis using the xCELLigence real-time cell analysis (RTCA) instrument (Agilent, Santa Clara, CA, USA) [32]. The cell index quantifies the impedance in electron flow caused by adhering cells, measured as resistance to an alternating current in arbitrary units. The percentage of cytolysis was calculated using the formula: Percentage of cytolysis = (cell index_no effector_– cell index_effector_) / (cell index_no effector_) × 100.

## Results

### Directed evolution of ICOS-L using yeast surface display for enhanced hICOS binding

**Fig. 1:**
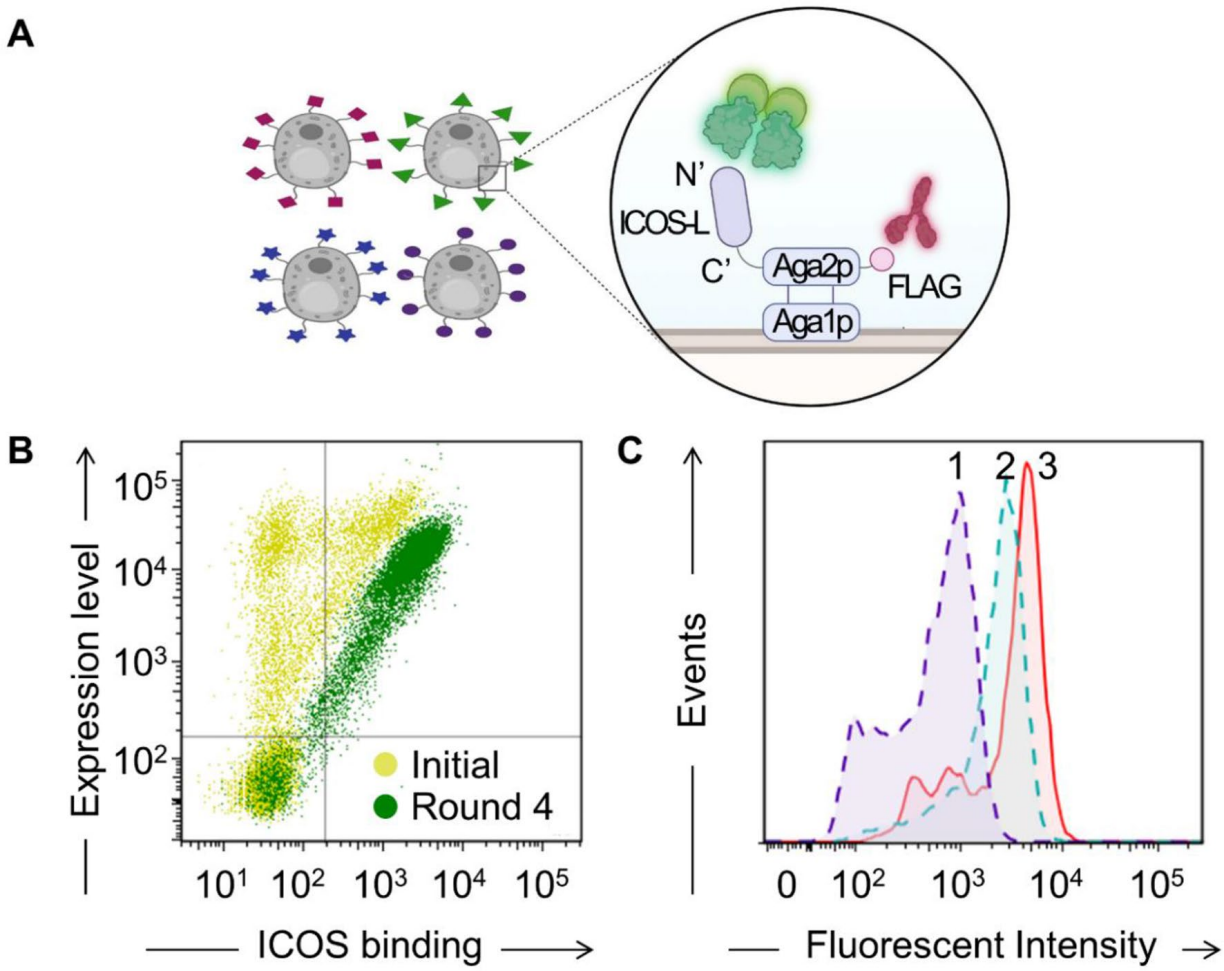
Isolation of ICOS-L variants with enhanced hICOS binding affinity. (**A**) Yeast surface display system for ICOS-L variants. (**B**) Density plot of yeast cells labeled with hICOS-Fc-Alexa488, showing the enrichment after rounds of FACS sorting. (**C**) FACS histogram displaying hICOS binding to yeast cells displaying wild-type ICOS-L (peak 1), A184 (peak 2), and Y8 (peak 3)

To enable the high-throughput screening of ICOS-L variants with enhanced affinity for hICOS, we first constructed a fluorescent hICOS detection probe by fusing the extracellular domain of hICOS to a human Fc domain, thereby mediating dimerization, and subsequently labeled the construct with Alexa Fluor 488. The purified hICOS-Fc probe exhibited strong binding to both wild-type ICOS-L and the A184 variant, one of several engineered ICOS-L clones previously reported by Levin et al. [24], thereby confirming the consistent biological activity and receptor engagement of the hICOS-Fc probe. A184 was selected based on its enhanced binding affinity, validated functional activity across multiple platforms, and favorable expression and stability characteristics, as demonstrated in the original study, which also reported its superior efficacy relative to other variants. To optimize a display configuration suitable for screening, we compared two yeast surface display formats in which ICOS-L was anchored either at the N-terminus or C-terminus of Aga2 (pCTCON-Aga2-ICOS-L-wild-type-FLAG and pCTCON-ICOS-L-wild-type-Aga2-FLAG, respectively). Consistent with structural insights indicating that the ICOS binding interface is localized to the N-terminus of ICOS-L (Supplementary Fig. 1A) [23], the C-terminal fusion format, which exposes the N-terminus, enabled significantly improved hICOS binding (Fig. 1A, Supplementary Fig. 1B).

Based on this configuration, we constructed a library of randomly mutated ICOS-L variants with a diversity exceeding 10⁷ unique sequences using the pCTCON-ICOS-L-wild-type-Aga2-FLAG vector. Dual-fluorescent labeling with anti-FLAG-iFluor647 and hICOS-Fc-Alexa488 enabled simultaneous monitoring of surface expression and hICOS binding. After four rounds of flow cytometry-based selection, the library became enriched for variants exhibiting both robust surface expression and increased binding to hICOS (Fig. 1B). Among the enriched clones, we selected Y8 as the lead candidate based on its strongest fluorescence signal in yeast display FACS analysis. Following mammalian expression and purification, Y8 exhibited the highest hICOS-binding affinity in ELISA, exceeding both wild-type ICOS-L and the previously reported A184 variant [24] (Fig. 1C, Supplementary Fig. 2).

### Synergistic interface remodeling by dual mutations enables 100-fold enhancement in ICOS-L binding to ICOS

**Fig. 2:**
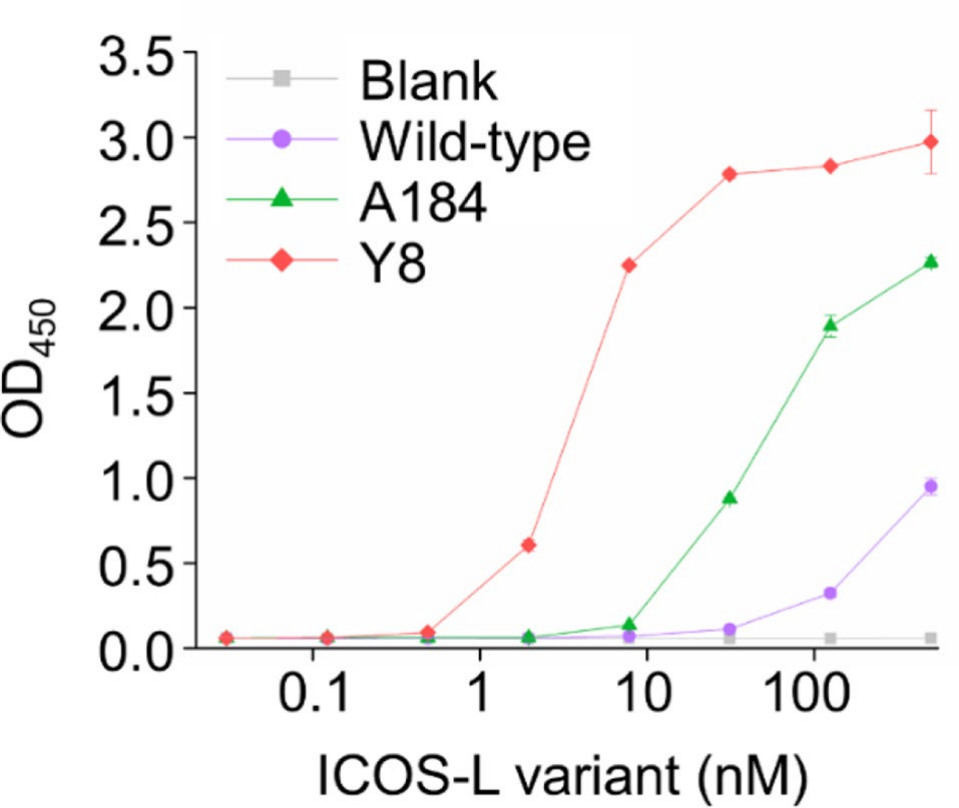
Evaluation of hICOS binding affinity to ICOS-L variants. ELISA graph showing hICOS binding to wild-type ICOS-L, A184, and Y8 variants

To assess the hICOS binding characteristics of the engineered ICOS-L variants, ICOS-L wild-type, A184, and Y8 (Q51P, N57H) were expressed and purified from mammalian cells. The binding affinity of these variants to hICOS was initially evaluated using ELISA, where the Y8 variant exhibited a significantly higher binding affinity to hICOS compared to A184 (Fig. 2). The quantitative binding kinetics and equilibrium dissociation constants (K_D_) of the ICOS-L variants were determined by bio-layer interferometry (BLI). To dissect the contribution of the individual Q51P and N57H mutations in Y8, expression constructs harboring either mutation alone (Q51P or N57H) were generated, expressed, and purified in mammalian cells alongside the ICOS-L wild-type, A184, and Y8 variants. All purified proteins were subjected to size exclusion chromatography, achieving high purity in all samples (Supplementary Fig. 3). BLI analysis demonstrated that the dual Q51P and N57H mutations in Y8 synergistically reduced the dissociation rate, resulting in a ~ 100-fold increase in hICOS binding affinity compared to wild-type ICOS-L and a 20-fold improvement over the A184 variant (Table 1, Supplementary Fig. 4). These findings establish Y8 as a functionally potent co-stimulatory agonist with superior receptor binding.

**Table 1:** Affinity constants of ICOS-L variants for hICOS, as determined by BLI analysis

	k_on_ (1/Ms)	k_off_ (1/s)	K_D_ (M)	Rank
Wild-type	2.98 × 10^5^	3.37 × 10^− 2^	1.13 × 10^− 7^	5
A184	2.80 × 10^4^	6.45 × 10^− 4^	2.31 × 10^− 8^	3
Y8	1.50 × 10^5^	1.79 × 10^− 4^	1.19 × 10^− 9^	1
N57H	2.44 × 10^5^	5.67 × 10^− 3^	2.33 × 10^− 8^	4
Q51P	2.02 × 10^5^	9.61 × 10^− 4^	4.75 × 10^− 9^	2

**Fig. 3:**
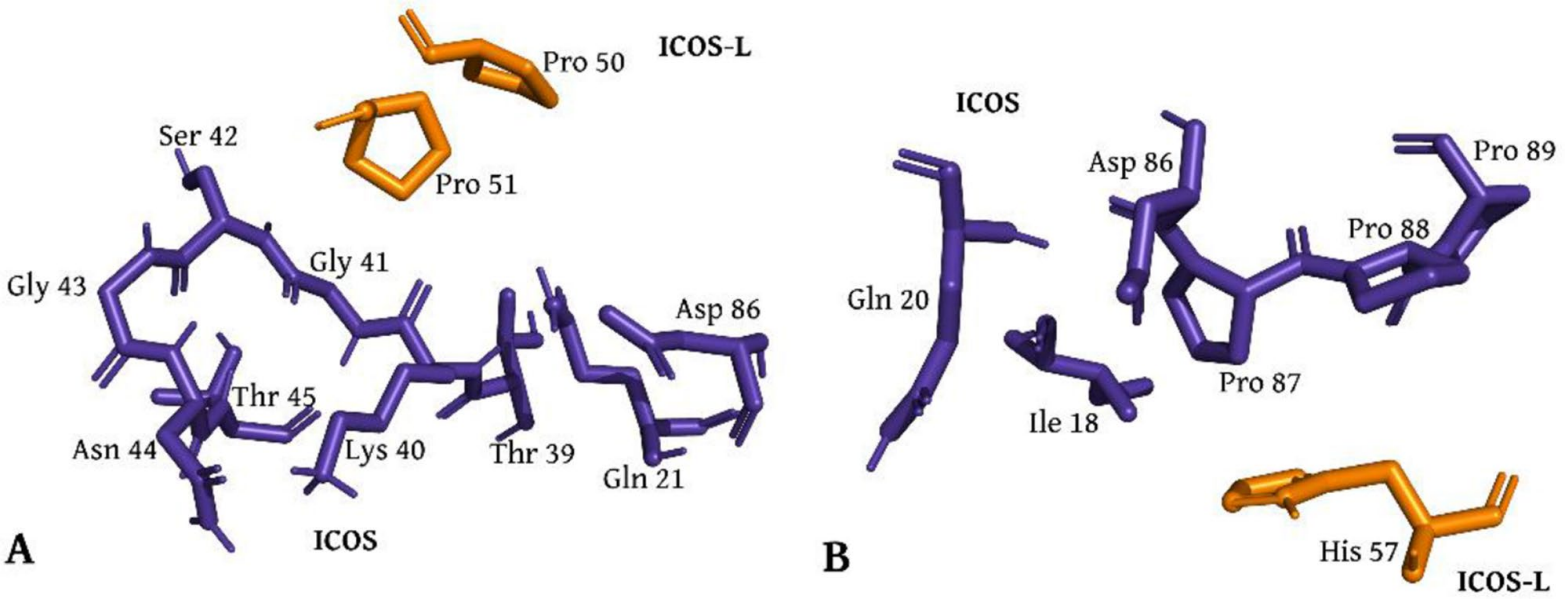
Residues of hICOS interacting with Pro51 and His57 of ICOS-L in the modeled Y8 mutant complex. (**A**) PPCheck analysis of the Q51P variant shows Pro51 forming van der Waals interactions with Gln21, Thr39, Lys40, Gly41, Ser42, Gly43, Asn44, Thr45, and Asp86. (**B**) PPCheck analysis shows His57 forming an electrostatic interaction with Asp86 and van derWaals interactions with Ile18, Gln20, Pro87, Pro88, and Pro89

To elucidate the molecular basis for this enhanced binding, a structural model of the Y8–hICOS complex was generated, and interfacial interactions were analyzed using PPcheck. The analysis revealed distinct and complementary interaction profiles conferred by each mutation (Fig. 3). Proline 51 (Pro51) introduced extensive van der Waals interactions with Gln21, Thr39, Lys40, Gly41, Ser42, Gly43, Asn44, Thr45, and Asp86 in hICOS (Fig. 3A), stabilizing the interface via hydrophobic packing. In parallel, histidine 57 (His57) interacted with Ile18, Gln20, Pro87, Pro88, and Pro89 through van der Waals forces and additionally formed a salt bridge with Asp86 (Fig. 3B), reinforcing the electrostatic complementarity at the binding surface. These structural insights provide a mechanistic explanation for the observed synergistic binding enhancement in Y8.

### Fc-fused Y8 variant potently activates T cells through enhanced hICOS binding

To evaluate the immunostimulatory potential of ICOS-L variants, we generated Fc fusion constructs of wild-type, A184, and Y8 ICOS-L proteins, each incorporating L234A/L235A/P329G (LALAPG) mutations to eliminate Fc-mediated effector functions [33]. These Fc-fused proteins were expressed in mammalian cells and purified to homogeneity. ELISA confirmed that the Y8-Fc fusion exhibited the strongest binding to hICOS among the tested variants (Supplementary Fig. 5), consistent with its engineered high-affinity interface.

**Fig. 4:**
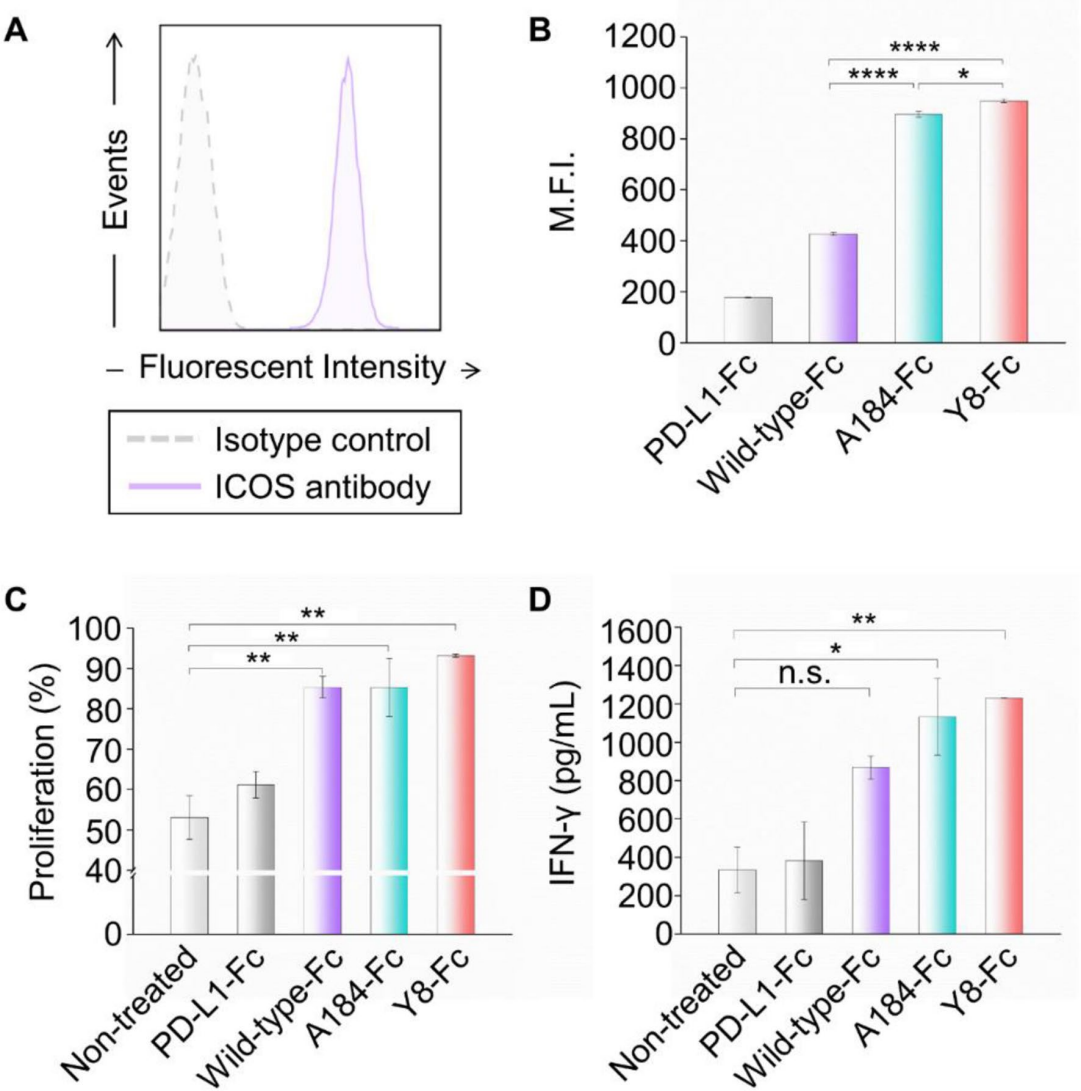
Characterization of Fc-fused ICOS-L variants. (**A**) FACS histograms for hICOS expression levels on activated T cells. (**B**) T cell binding analysis of Fc-fused ICOS-L variants by FACS at 200 nM protein concentration (M.F.I.: mean fluorescence intensity). (**C** and **D**) Functional evaluation of T cell co-stimulation by Fc-fused ICOS-L variants at 50 nM, measuring (**C**) T cell proliferation and (**D**) IFN-γ production

To functionally assess T cell engagement, primary human T cells were freshly isolated from peripheral blood mononuclear cells (PBMCs) and activated using anti-CD3/anti-CD28 stimulation. Flow cytometry verified robust induction of hICOS expression on the activated T cells (Fig. 4A). Subsequent binding analysis revealed that the Y8-Fc fusion exhibited superior binding to hICOS on the surface of activated T cells, surpassing the wild-type and A184 variants (Fig. 4B). Binding was initially tested at both 20 and 200 nM, and a clear signal was only detectable at 200 nM, which was subsequently used for comparison. Next, we evaluated the functional consequence of hICOS engagement by measuring T cell proliferation and cytokine secretion. Although 200 nM was initially used based on binding data, this concentration produced saturated activation signals, limiting the ability to distinguish between samples. Therefore, we lowered the concentration to 50 nM for subsequent assays to enhance resolution of functional differences. Treatment with Fc-fused ICOS-L variants resulted in increased T cell activation relative to the negative control (PD-L1-Fc). Among the tested constructs, the Y8 variant induced the most robust T cell proliferation and significantly elevated IFN-γ secretion, a hallmark of effector T cell function (Fig. 4C and D). These findings establish Y8 as a functionally potent co-stimulatory agonist with superior receptor binding.

### Modular Y8 fusion to pembrolizumab enables dual engagement of PD-1 and hICOS

To explore the potential of the Y8 variant as a fusion partner for enhancing the efficacy of immune checkpoint inhibitors (ICIs), we generated fusion constructs in which engineered ICOS-L Y8 was incorporated into a modular checkpoint fusion format. Specifically, we fused Y8 to pembrolizumab, a clinically approved anti–PD-1 IgG4, using our in-house engineered Fc backbone (Fc-SH) designed to silence Fc effector functions and extend serum half-life. As expected, pembrolizumab (Fc-SH) completely abolished binding to Fcγ receptors while markedly enhancing binding to human FcRn at pH 6.0, relative to the conventional IgG4 S228P backbone (Supplementary Fig. 6).

**Fig. 5:**
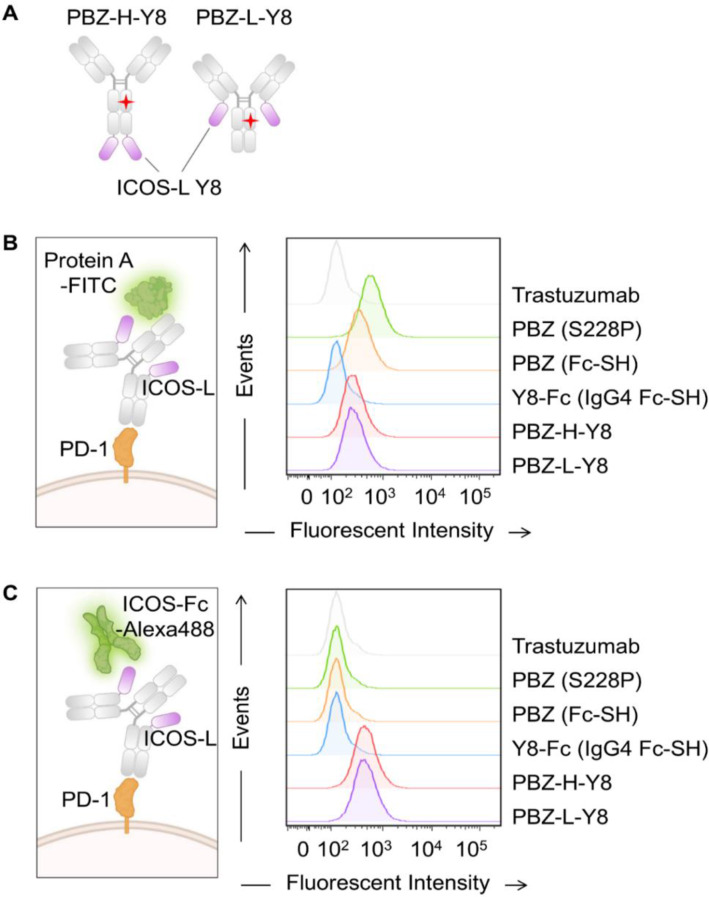
Generation of Pembrolizumab (Fc-SH)-fused Y8. (**A**) Schematic illustration of PBZ-H-Y8 and PBZ-L-Y8, showing the Y8 variant fused to the heavy chain (PBZ-H-Y8) and light chain (PBZ-L-Y8) of pembrolizumab. (**B** and **C**) FACS histograms showing (**B**) cell surface PD-1 binding and (**C**) simultaneous cell surface PD-1/soluble hICOS dual binding, both assessed at a protein concentration of 100 nM

Expression constructs were designed to fuse Y8 to the C-terminus of either the heavy or light chain of pembrolizumab (Fc-SH), and the resulting fusion proteins were expressed in mammalian cells with comparable yields to unmodified pembrolizumab (Fig. 5A). ELISA analysis confirmed that Y8 fusion did not impair PD-1 binding, while significantly improving ICOS binding compared to pembrolizumab fused to wild-type ICOS-L (Supplementary Fig. 7A and B). Dual-targeting capability was verified by bridging ELISA, which demonstrated simultaneous and stable engagement of both PD-1 and hICOS by the purified Y8 fusion constructs (Supplementary Fig. 7C).

To further validate the cell-surface binding of these constructs, PD-1-overexpressing CHO cells were incubated with pembrolizumab-Y8 fusions at a concentration of 100 nM. Although the PD-1 binding of pembrolizumab-Y8 was slightly lower than that of pembrolizumab alone, robust PD-1 recognition was maintained. Notably, fluorescence-based dual-color binding assays confirmed that pembrolizumab-Y8 simultaneously binds to both surface PD-1 and hICOS, establishing the bifunctionality of the modular fusion design. These results highlight the versatility of the Y8 fusion format in enabling dual checkpoint targeting while preserving essential binding characteristics of pembrolizumab (Fig. 5B and C).

### Engineered ICOS-L Y8 fusion potentiates checkpoint blockade through a modular design for immune activation and tumor clearance

To evaluate the immunostimulatory capacity of Y8-based constructs, we first compared T cell activation in response to Fc-fused ICOS-L variants and pembrolizumab-Y8 fusion proteins. Among the ICOS-L variants, Y8-Fc (Fc-SH) elicited the strongest T cell activation, as evidenced by elevated proliferation and cytokine release. While the pembrolizumab-Y8 fusion construct exhibited slightly attenuated activity relative to Y8-Fc alone, it still significantly outperformed the parental pembrolizumab (Fc-SH), demonstrating that fusion with Y8 effectively retained hICOS co-stimulatory activity within the checkpoint blockade context (Supplementary Fig. 8).

**Fig. 6:**
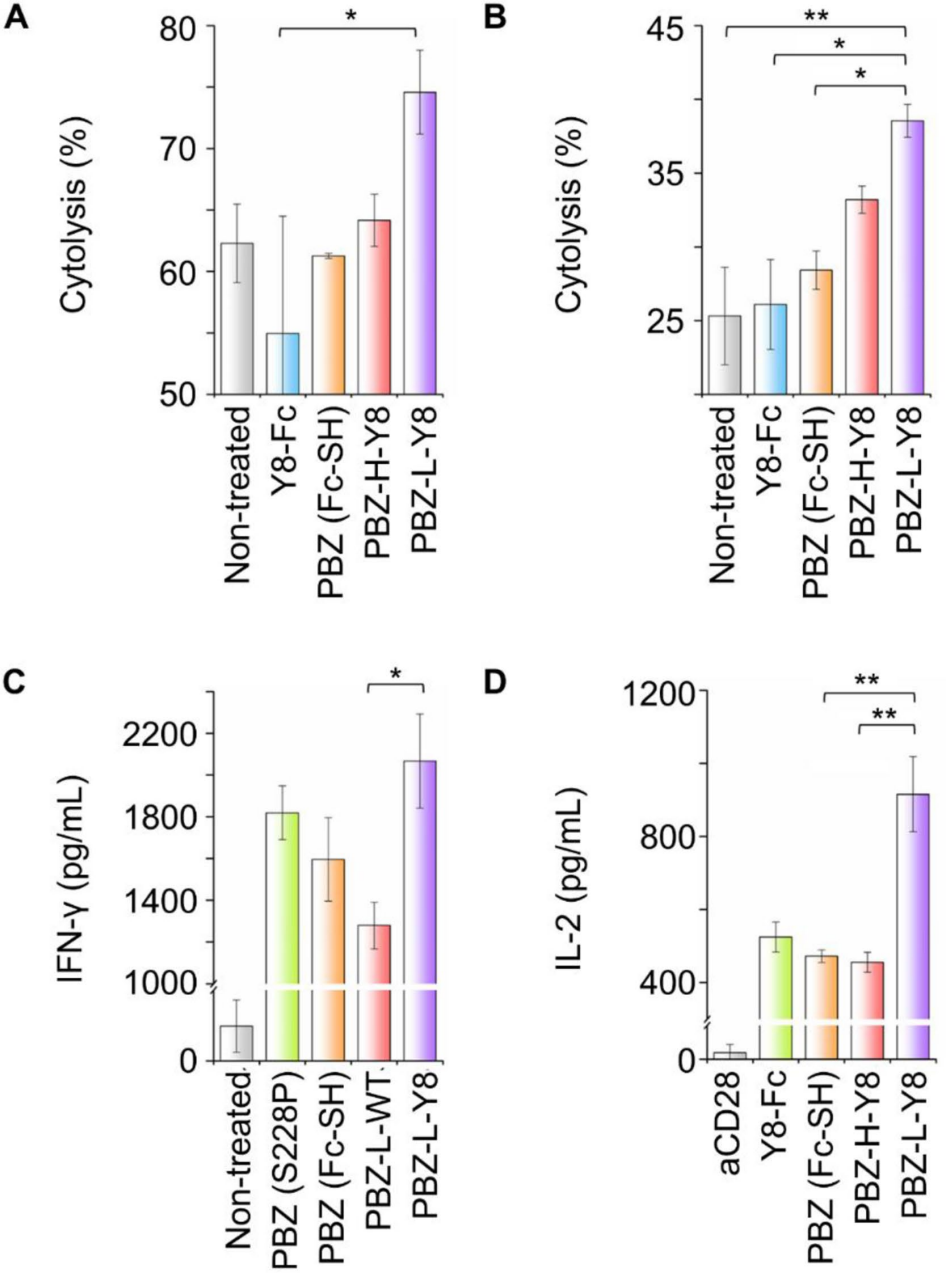
Evaluation of T cell activation by Pembrolizumab-Y8. (**A** and **B**) T cell-mediated tumor lysis induced by Pembrolizumab-Y8 at a protein concentration of 2 nM and an effector-to-target (E: T) ratio of 1:2. (**A**) A375 cells. (**B**) MDA-MB-231 cells. (**C** and **D**) Cytokine production analysis in mixed lymphocyte reaction (MLR) assays treated with Pembrolizumab-L-ICOS-L at 200 nM. (**C**) IFN- ɣ production. (**D**) IL-2 production. Y-axis scales were individually adjusted to highlight sample-specific patterns. Corresponding raw data with standardized axes are provided in Supplementary Fig. 9

We next assessed the antitumor potential of pembrolizumab-Y8 fusion proteins in real-time T cell–mediated cytotoxicity assays against PD-L1-overexpressing tumor cell lines, MDA-MB-231 and A375. To prevent signal saturation and facilitate construct discrimination, we used 2 nM protein and an effector-to-target (E: T) ratio of 1:2. Both pembrolizumab-H-Y8 (heavy chain fusion) and pembrolizumab-L-Y8 (light chain fusion) markedly enhanced tumor cell lysis compared to pembrolizumab alone, underscoring the therapeutic advantage of integrating ICOS-mediated co-stimulation with PD-1 blockade (Fig. 6A and B). Notably, pembrolizumab-L-Y8 induced the highest levels of IFN-γ and IL-2 secretion, key indicators of T cell activation and effector function. Cytokine assays were conducted at 200 nM, a concentration selected to maximize signal strength while avoiding saturation, thereby demonstrating that light chain fusion of Y8 optimally balances PD-1 blockade with ICOS engagement (Fig. 6C and D). To improve data transparency and account for differences in Y-axis scaling, the corresponding raw data with unified axes are also provided (Supplementary Fig. 9).

To further support the translational applicability of the Y8 variant, we examined its cross-reactivity with mouse ICOS and confirmed that it maintained high-affinity binding, enabling preclinical in vivo evaluation using murine models (Supplementary Fig. 10). Taken together, these results position ICOS-L Y8 as a modular immunostimulatory component that synergizes with PD-1 blockade to elicit potent T cell activation and tumor clearance, offering a promising framework for next-generation cancer immunotherapies.

## Discussion

Modular design and protein engineering strategies are increasingly pivotal in overcoming the limitations of immune checkpoint inhibitors (ICIs), particularly in amplifying co-stimulatory signaling and overcoming resistance. In this study, we engineered the ectodomain of ICOS-L to significantly enhance its binding affinity to hICOS, generating a potent agonist capable of robustly stimulating T cell activation. Through yeast surface display–based directed evolution, we identified an ICOS-L variant (Y8) carrying two synergistic mutations (Q51P and N57H) that enhanced hICOS affinity by ~ 100-fold relative to wild-type, leading to markedly increased T-cell activation in both in vitro assays and tumor lysis models. When fused to pembrolizumab, an anti–PD-1 antibody, the Y8 variant promoted superior anti-tumor responses compared to pembrolizumab alone, demonstrating that the addition of ICOS co-stimulation enhances the efficacy of PD-1 blockade. This finding highlights the potential of combining checkpoint inhibition with co-stimulatory agonism to improve therapeutic responses in settings where anti–PD-1 monotherapy provides limited benefit.

The enhanced performance of Y8 stems from two point mutations strategically positioned within the IgV domain of ICOS-L, the principal receptor-binding interface. Compared to wild-type and the previously reported A184 variant [24], Y8 exhibited markedly improved affinity, largely driven by a decreased dissociation rate. Structural modeling revealed that although proline residues are typically associated with hydrophobic interactions [34–36], the Q51P mutation in Y8 likely exerts its effect through conformational stabilization by promoting local rigidity. The N57H mutation further complements this interaction by forming an electrostatic contact with Asp86 of hICOS. These findings illustrate how targeted mutations can reconfigure binding energetics and geometry to produce high-affinity receptor engagement. While enhancing affinity is a common goal in protein engineering, it does not inherently guarantee improved therapeutic efficacy [37]. To address this, we systematically compared ICOS-L variants with distinct binding affinities in functional assays. The high-affinity Y8 variant consistently demonstrated superior agonistic activity, particularly in the context of the PD-1–ICOS bispecific fusion format. These findings indicate that, within this therapeutic framework, affinity maturation can translate into meaningful functional gains, despite the broader recognition that affinity alone is not always predictive of efficacy.

Functionally, Y8 potently activated T cells when immobilized, consistent with prior observations that plate-bound ICOS-L variants act as agonists [24]. Interestingly, while soluble ICOS-L variants displayed antagonistic behavior in isolation, fusion to pembrolizumab converted Y8 into a potent agonist. This functional shift likely reflects antibody-mediated co-localization of ICOS and PD-1 on the same T cell, enabling receptor clustering required for downstream activation. These results underscore how structural integration of immunomodulatory ligands into antibody scaffolds can transform context-sensitive activity into robust co-stimulatory signaling.

The fusion of Y8 to pembrolizumab enhanced T cell–mediated tumor cell killing, whereas fusion to atezolizumab (anti–PD-L1), which engages its target in trans, failed to improve cytotoxicity (Supplementary Fig. 11). This contrast reveals that co-delivery of ICOS agonism and PD-1 blockade to the same immune cell is a critical determinant of synergy, offering mechanistic guidance for future modular or bispecific immunotherapeutic constructs [38]. Fusion geometry further influenced efficacy. The light-chain fusion format (pembrolizumab-L-Y8) significantly outperformed heavy-chain fusion in both tumor lysis and cytokine production, likely due to favorable spatial orientation of the ICOS-L domain relative to cell-surface ICOS. This highlights the importance of architectural considerations when designing fusion-based therapeutics, particularly in terms of receptor accessibility and signal integration. Given these considerations, antibody fragment–based formats, such as Fab or scFv fusions, represent a promising strategy to reduce molecular size and enhance tissue distribution. These designs may be particularly advantageous in tumors with limited vascularization or dense stromal architecture. Nonetheless, shortened serum half-life and structural instability remain key challenges requiring additional optimization. Although we used full-length antibodies in this study to ensure clinical relevance, fragment-based designs merit future exploration to further expand the applicability of this modular platform.

Beyond oncology, ICOS-L variants exhibit molecular format–dependent functional plasticity. For instance, ICOS is overexpressed on intratumoral regulatory T cells (Tregs), and soluble ICOS-L variants could potentially deplete these suppressive cells [39, 40]. Conversely, in autoimmune settings, engineered ICOS-L may serve to attenuate aberrant T cell activation and restore immune homeostasis [41, 42]. Such application-specific flexibility positions ICOS-L as a versatile synthetic platform for immune modulation suitable for therapeutic adaptation across cancer, autoimmunity, and inflammatory disorders [43–50].

## Conclusions

Our directed evolution strategy yielded ICOS-L variants with markedly improved hICOS binding affinity via minimal mutations. These results highlight the broader applicability of structure-informed evolution for tuning receptor-ligand interactions in co-stimulatory pathways. The Y8 variant, characterized by superior receptor affinity and potent T cell activation, demonstrates the translational potential of ICOS-L engineering in immune-oncology. Moreover, its success in modular antibody fusion formats underscores its therapeutic versatility, supporting its further development as a synthetic immunomodulatory component. Together, these findings define engineered ICOS-L variants as a programmable platform for next-generation immune intervention.

## Electronic supplementary material

Below is the link to the electronic supplementary material.

Supplementary Material 1

## Data Availability

No datasets were generated or analysed during the current study.
