# Supplementary material for: Directed evolution and modular integration of a high-affinity ICOS-L variant for potent T cell–mediated tumor elimination
Source: J Biol Eng. 2025 Jul 11;19:63. doi: 10.1186/s13036-025-00536-6 (PMC12255069; doi:10.1186/s13036-025-00536-6)
Supplement: Supplementary file 1 — Supplementary Material 1 [file 13036_2025_536_MOESM1_ESM.docx]

**SUPPLEMENTARY INFORMATION**

**Directed Evolution and Modular Integration of a High-Affinity ICOS-L Variant for Potent T Cell–Mediated Tumor Elimination**

Ji Yeon Ha^1,2^, Tae Wook Song^3^, Petrina Jebamani^4^, Sun-Gu Lee^4^, Sang Taek Jung^3,5,6,7,8*^

*^1^Department of Biomedical Sciences, Graduate School, Korea University, Seoul 02841, Republic of Korea*

*^2^BK21 Graduate Program, Department of Biomedical Sciences, Korea University College of Medicine, Seoul 02841, Republic of Korea*

*^3^Department of Chemical and Biological Engineering, Seoul National University, Seoul 08826, Republic of Korea*

*^4^Department of Chemical Engineering, Pusan National University, Busan, 46241, Republic of Korea*

*^5^Interdisciplinary Program for Bioengineering, Seoul National University, Seoul 08826, Republic of Korea*

*^6^Institute of Chemical Processes, Seoul National University, Seoul 08826, Republic of Korea*

*^7^BioMAX, Seoul National University, Seoul 08826, Republic of Korea*

*^8^Seoul National University Medical Research Center (SNUMRC), Seoul 03080, Republic of Korea*

***Correspondence**: *Sang Taek Jung (stjung@snu.ac.kr)

**Supplementary Figures**

**Fig. S1 Selection of display system. (A)** Structure of the ICOS/ICOS-L complex (PDB: 6X4G). **(B)** FACS analysis showing hICOS binding to yeast surface displaying ICOS-L wild-type.

**
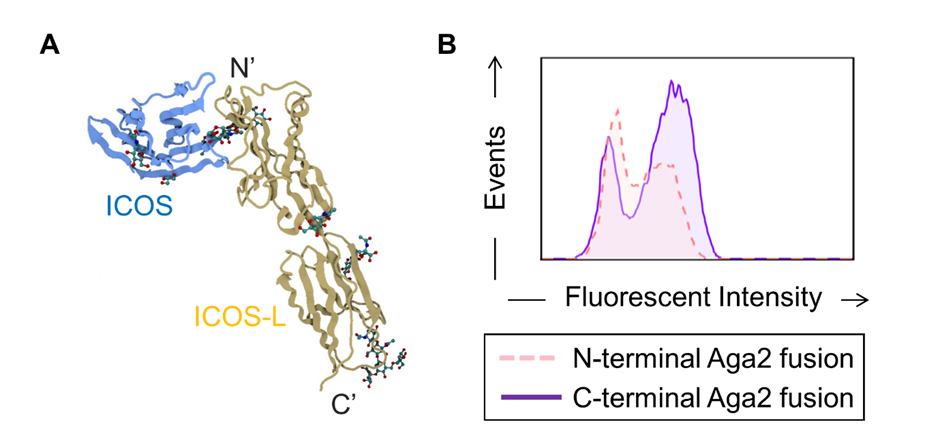
**

**Fig. S2 Amino acid sequence alignment of ICOS-L variants compared to wild-type ICOS-L.**
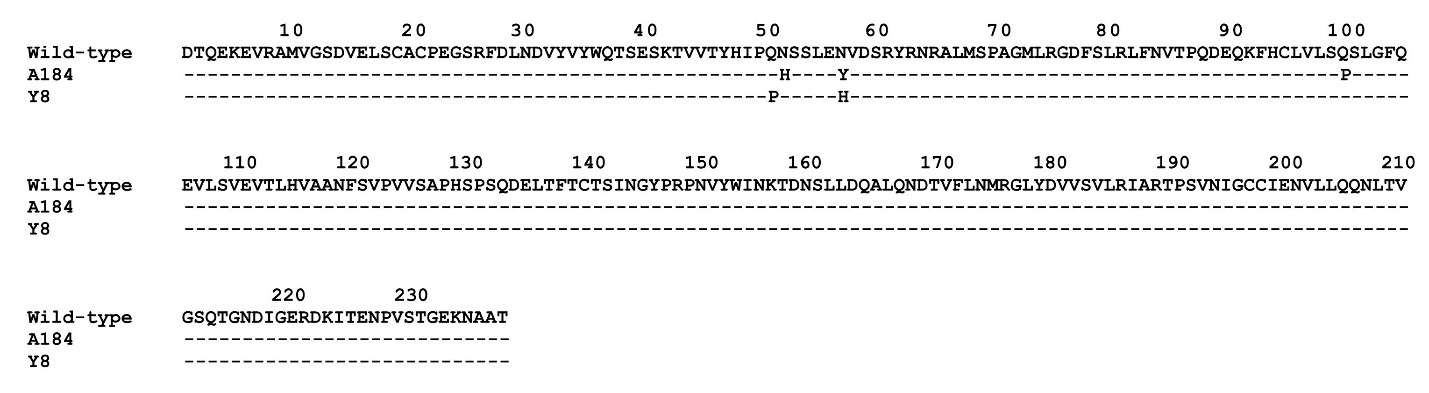


**Fig. S3 Size exclusion chromatography profile for the purification of ICOS-L variants.**

**
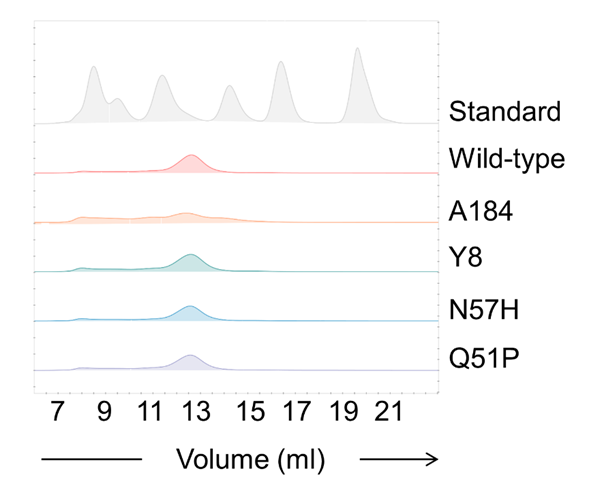
**

**Fig. S4 Bio-layer interferometry analysis for ICOS-L variants.** Binding to immobilized hICOS-Fc on Protein A biosensors was measured. Sensorgrams and kinetic fits are shown for: **(A)** Wild-type, **(B)** A184, **(C)** Y8, **(D)** N57H, **(E)** Q51P

**
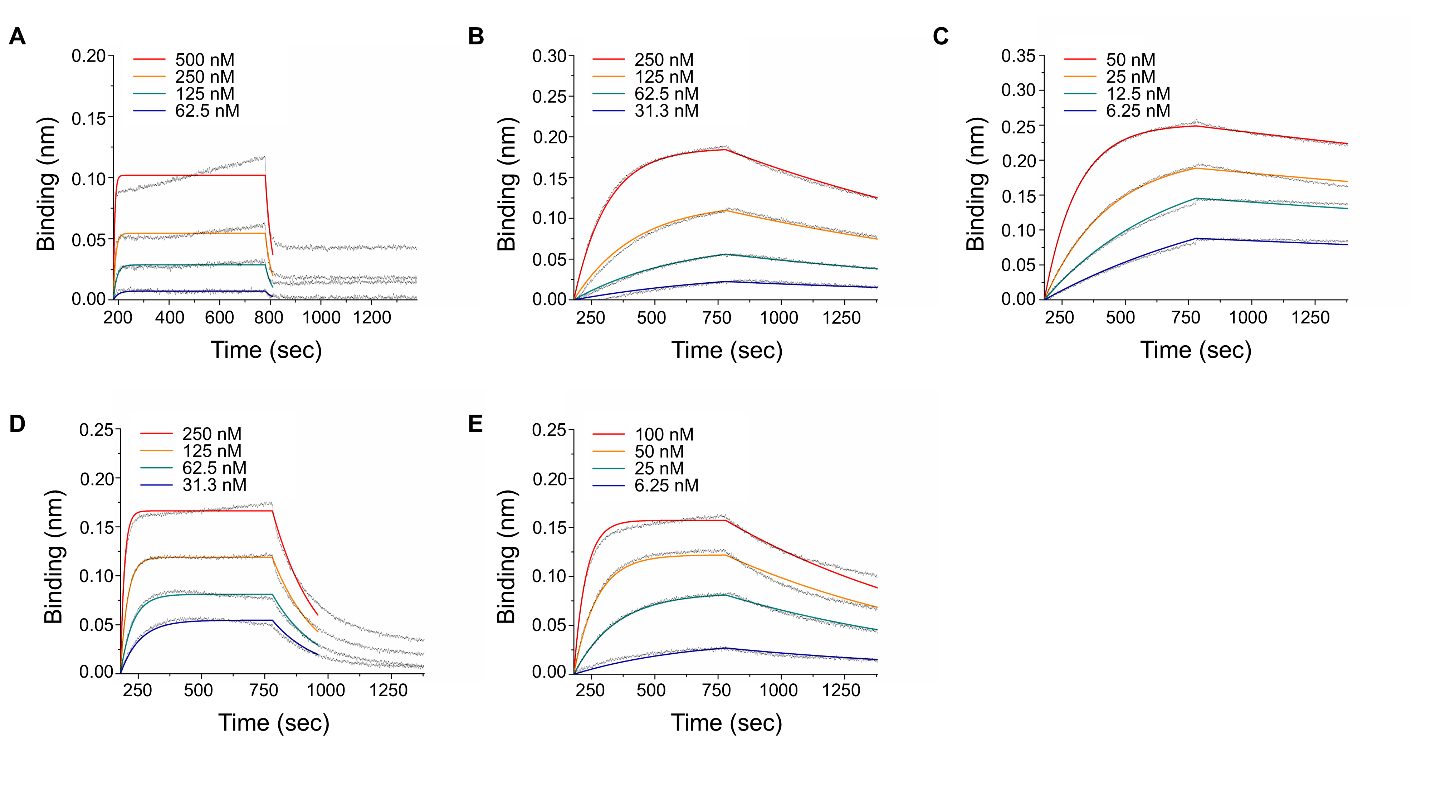
**

**Fig. S5 ELISA analysis of hICOS binding of ICOS-L variants fused with Fc.**


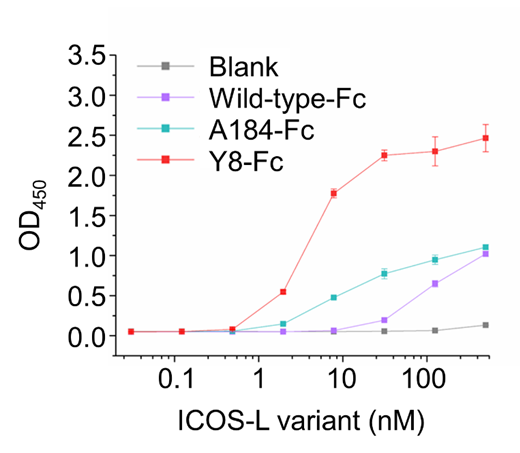


**Fig. S6** **Generation of pembrolizumab with Fc mutations. (A–H)** ELISA analysis of binding to **(A)** FcγRI, **(B)** FcγRIIa-H, **(C)** FcγRIIa-R, **(D)** FcγRIIb, **(E)** FcγRIIIa-V, **(F)** FcγRIIIa-F, **(G)** FcRn at pH 6.0, and **(H)** FcRn at pH 7.4.


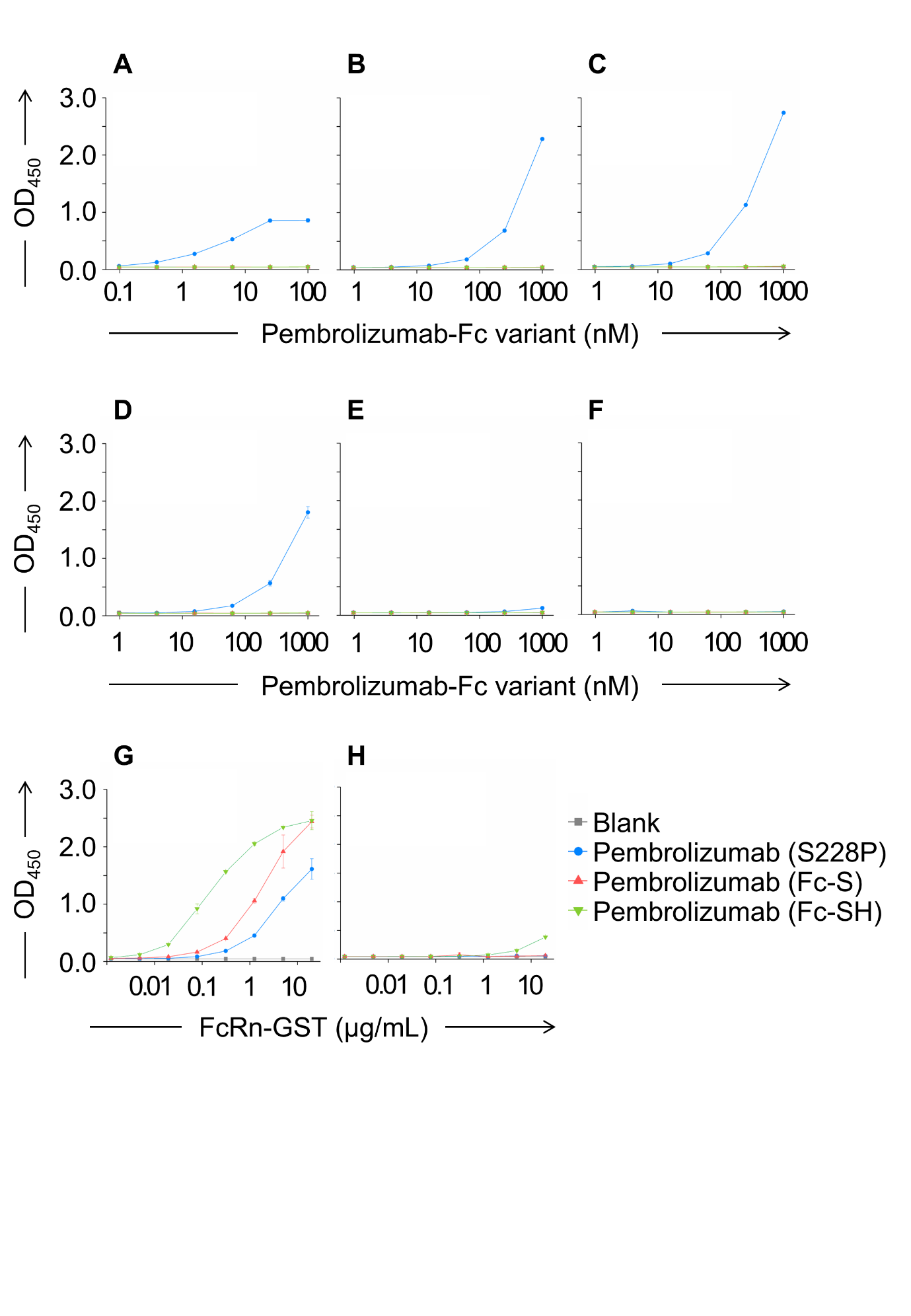


**Fig. S7** **Binding analysis of fusion proteins by ELISA.** (**A**) PD-1 binding. (**B**) hICOS binding. (**C**) Dual binding of PD-1 and hICOS.


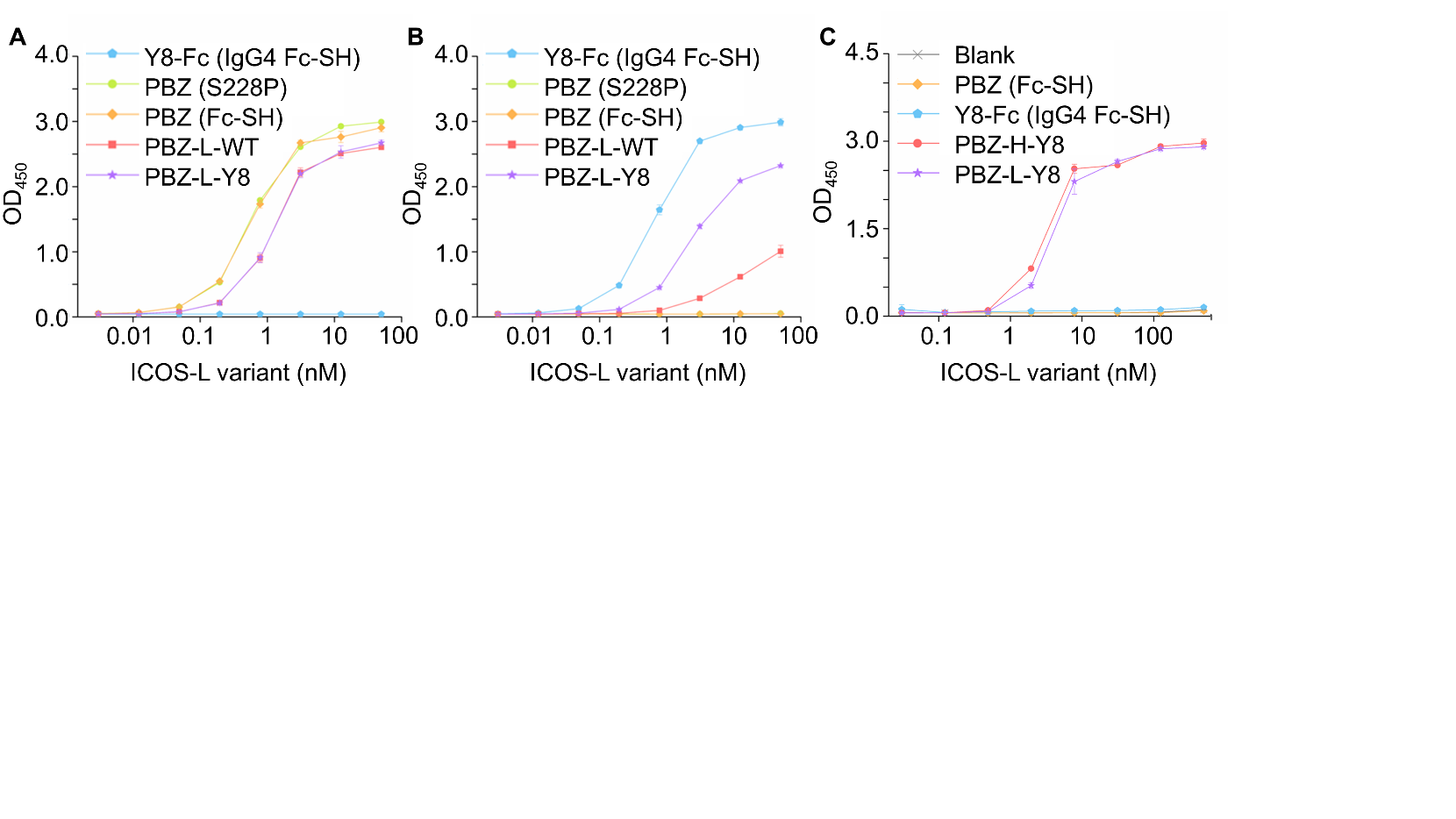


**Fig. S8** **Evaluation of T cell co-stimulation by fusion proteins.** **(A)** Proliferation of CD4+ T cells. **(B)** Proliferation of CD8+ T cells.


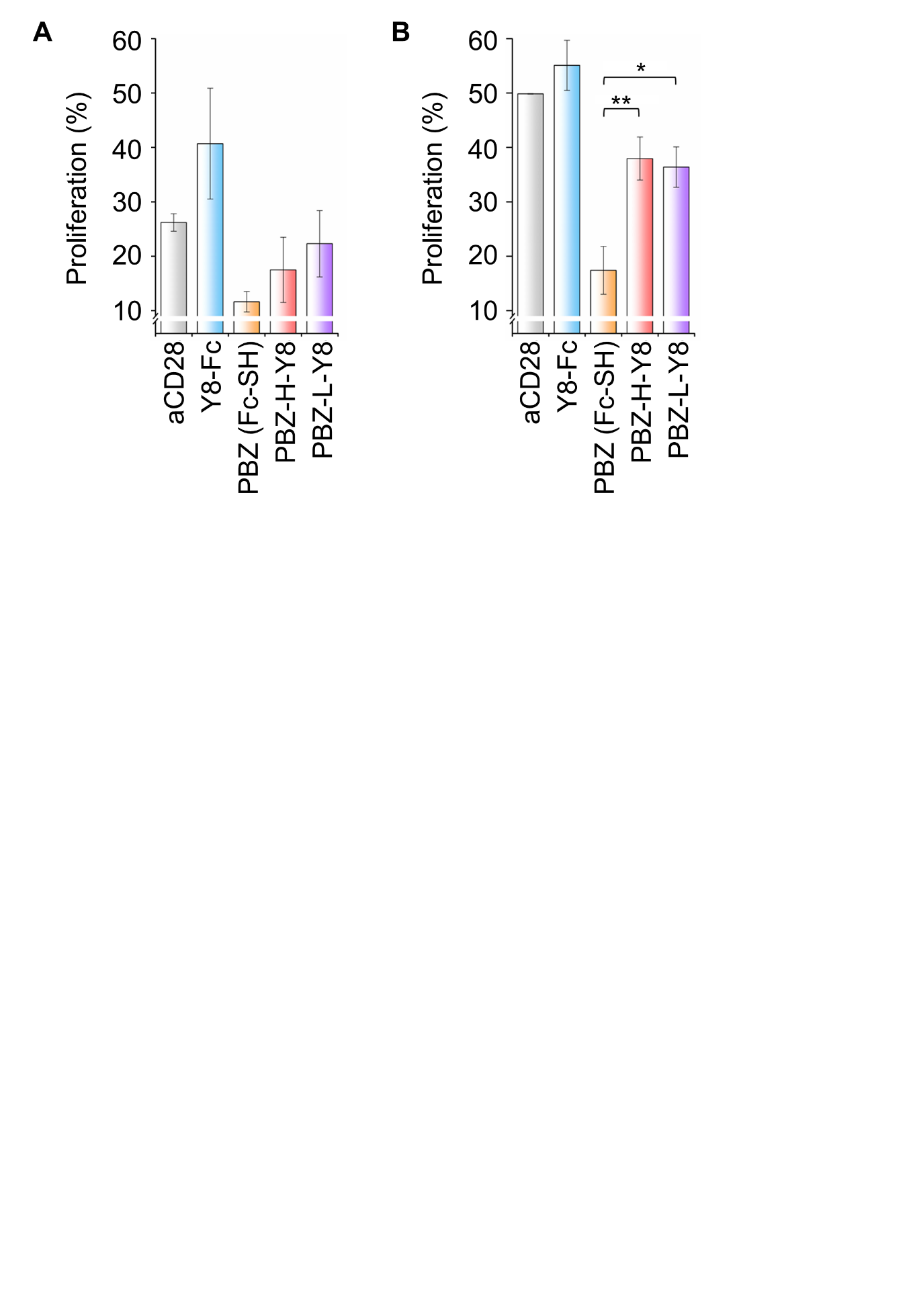


**Fig. S9** **Raw data corresponding to Fig. 6 with unified Y-axis scaling. (A** and **B)** T cell-mediated tumor lysis induced by Pembrolizumab-Y8. (**A)** A375 cells. **(B)** MDA-MB-231 cells. **(C** and **D)** Cytokine production analysis in mixed lymphocyte reaction (MLR) assays treated with Pembrolizumab-L-ICOS-L. **(C)** IFN- ɣ production. **(D)** IL-2 production.

**
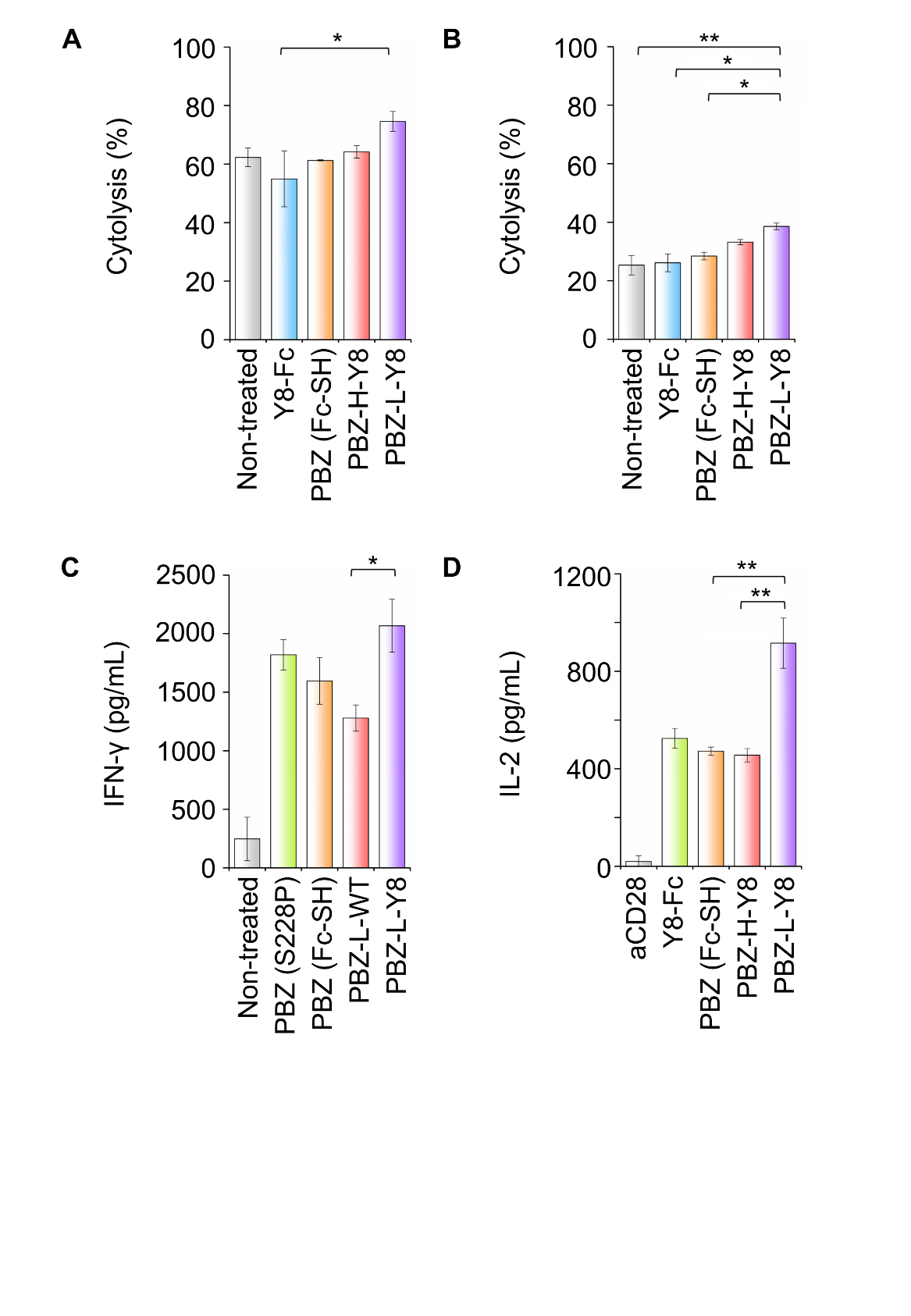
**

**Fig. S10** **ELISA analysis of the binding affinity of mouse ICOS to pembrolizumab-L-ICOS-L.**


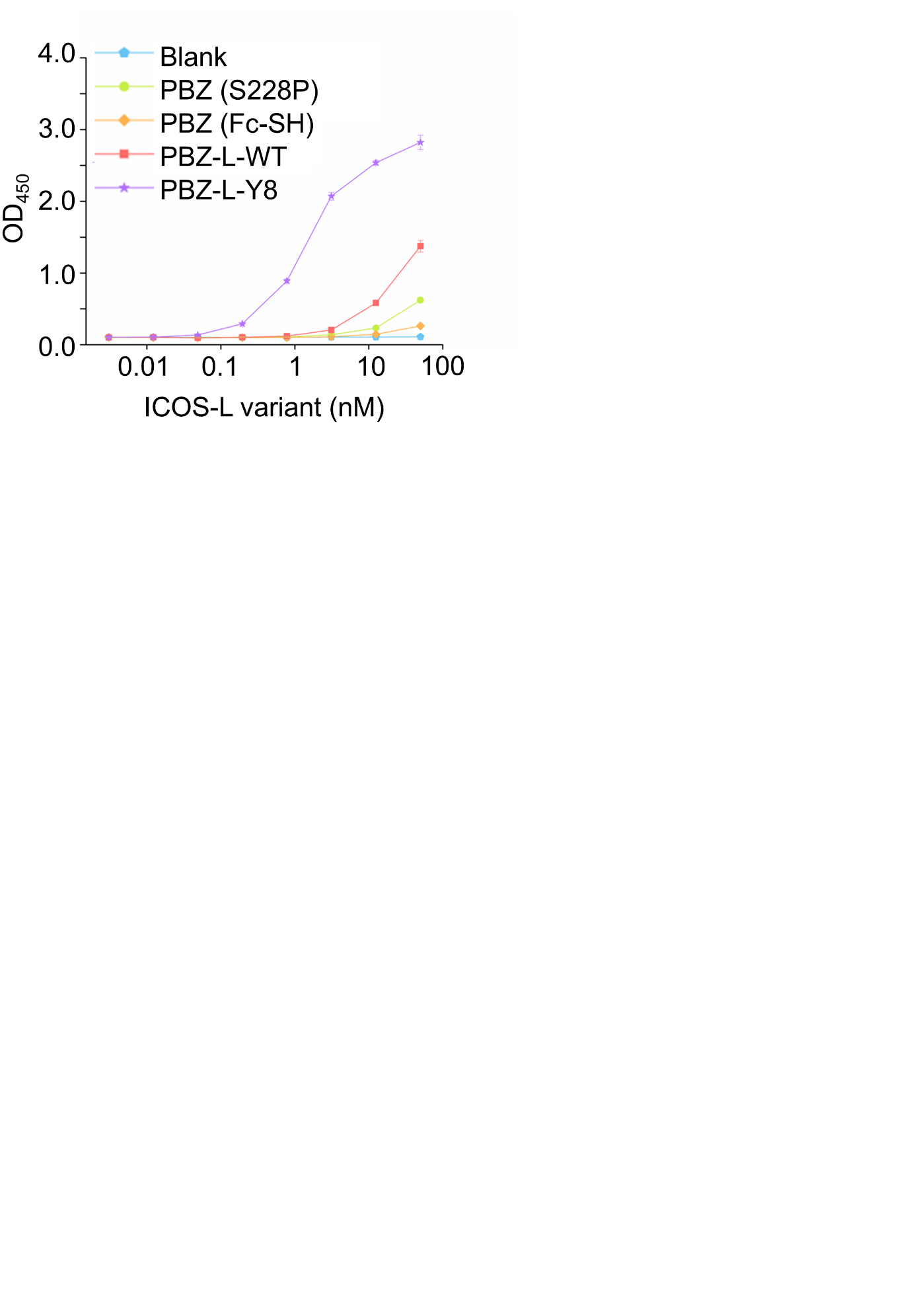


**Fig. S11** **Evaluation of T cell-mediated tumor (MDA-MB-231) lysis induced by atezolizumab (ATZ)-Y8.**

**
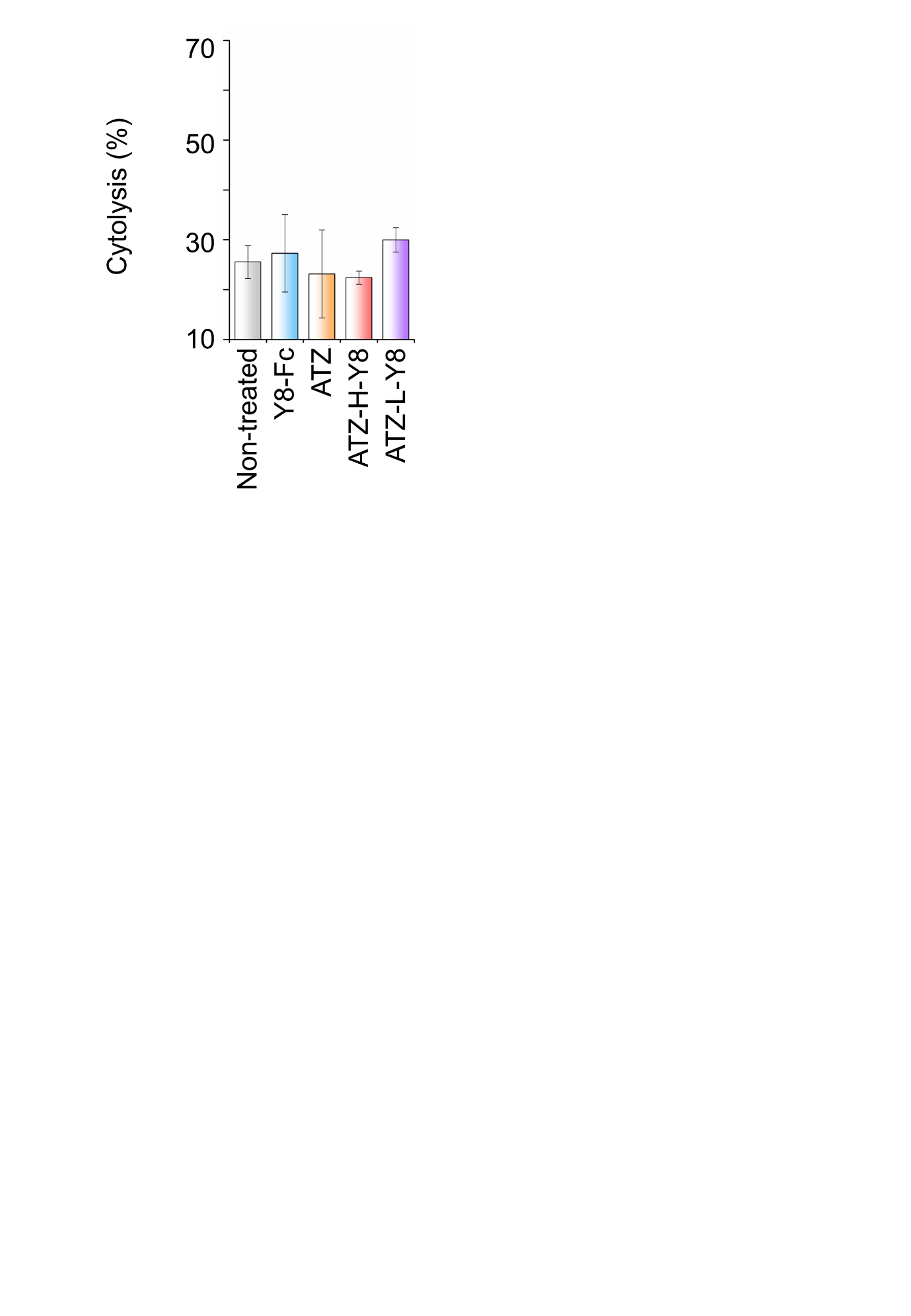
**

**Supplementary Tables**

**Table S1.** List of oligonucleotide primers used in this study.

| primer Name | sequence (5’→3’) |
| --- | --- |
| JYI#1 | GCGGAATTCGGCGCGCACTCCGAGATCAACGGCAGCGCCAATTAC |
| JYI#3 | GCCCTTAATTTTCCAATAACCTAGTATAGGGGACATAGAGCCACCCTTCAACTGACAACAGAGCTGGCTCTC |
| JYI#4 | CATGTGTGAGTTTTGTCACAAGATTTGGGAGAGCCACCCTTCAACTGACAACAGAGCTGGCTCTC |
| JYI#5 | CCCAAATCTTGTGACAAAACTCACACATG |
| JYI#6 | GCGGAATTGGCGCGCACTCCGACACCCAGGAAAAGGAGGTCCG |
| JYI#7 | GAATTCCGCTCTAGATTATCAATGATGATGGTGGTGATGACTACCTCCAGTCGCGGCGTTCTTCTCG |
| JYI#8 | CGCAGCGAGGCCCAGCCGGCCGACACCCAGGAAAAGGAGGTCCG |
| JYI#9 | CGCAGCGA GGCCCCCGAGGCCCCAGTCGCGGCGTTCTTCTCG |
| JYI#14 | ATCCACTTTGCCTTTCTCTCCACAGGCGCGCACTCCGACACCCAGGAAAAGGAGGTCCG |
| JYI#15 | CAGCGGGTTTAAACGGGCCCTCTAGATTATCAATGATGATGGTGGTGATGACTACCTCCAGTCGCGGCGTTCTTCTCG |
| JYI#16 | CTGTGGTAACCTACCACATCCCCCAGAATAGCAGTCTAGAACACGTCGACAGC |
| JYI#17 | GCTGTCGACGTGTTCTAGACTGCTATTCTGGGGGATGTGGTAGGTTACCACAG |
| JYI#18 | CACATCCCCCCGAATAGCAGTCTAGAAAACGTCGACAGCCGGTATAGGAAC |
| JYI#19 | GTTCCTATACCGGCTGTCGACGTTTTCTAGACTGCTATTCGGGGGGATGTG |
| JYI#21 | GAATTCCGCTCTAGATTATCAATGATGATGGTGGTGATGACTACCTCCGTGCAGGTAGCCCCCAGTCAG |
| JYI#23 | CGGTGGGCATGTGTGAGTTTTGTCGGATCCACCAGTCGCGGCGTTCTTCTCG |
| JYI#24 | GACAAAACTCACACATGCCCACCG |
| JYI#25 | GAATTCCGCTCTAGATTATCATTTACCCGGGGACAGGGAGAG |
| JYI#40 | CAGCGGGTTTAAACGGGCCCTCTAGATTATCATTTACCCGGAGACAGGGAGAGG C |
| JYI#46 | GCGGAATTCGGCGCGCACTCCCAGGTGCAGCTGGTGCAGAGC |
| JYI#47 | CACTGTCAGCACGGACACCACG |
| JYI#48 | GTGGTGTCCGTGCTGACAGTGTATCACATGGATTGGCTGAACGGCAAGGAGTATAAG |
| JYI#49 | CAGGCTTTTCTGAGTGTAATGGTTGTGCAGTGCTTCGTGCAGCACGCTACAGCTGAACACGTTGC |
| JYI#50 | GAATTCCGCTCTAGATCACTTGCCCAGGGACAGTGACAGGCTTTTCTGAGTGTAATGGTTGTGC |
| JYI#55 | GCTCCCTCCACCACCACTGCCTCCACCCCCTGATCCCCCACCGCCCTTGCCCAGGGACAGTGACAGG |
| JYI#56 | GCAGTGGTGGTGGAGGGAGCGACACCCAGGAAAAGGAGGTCCG |
| JYI#57 | TCTAGATCAAGTCGCGGCGTTCTTCTCG |
| JYI#58 | ATCCACTTTGCCTTTCTCTCCACAGGCGCGCACTCCCAGGTGCAGCTGGTGCAGAGC |
| JYI#59 | CAGCGGGTTTAAACGGGCCCTCTAGATCAAGTCGCGGCGTTCTTCTCG |
| JYI#60 | GAGATCGTGCTGACCCAGAGCC |
| JYI#61 | GCTCCCTCCACCACCACTGCCTCCACCCCCTGATCCCCCACCGCCGCACTCGCCCCTGTTGAAGC |
| JYI#62 | ATCCACTTTGCCTTTCTCTCCACAGGCGCGCACTCCGAGATCGTGCTGACCCAGAGCC |
| JYI#77 | CCACAGGCGCGCACTCC GACACCCAGGAAAAGGAGGTCCG |
| JYI#78 | CAGGGTGGTCCGTACTTGCTCTCGGATCCACCAGTCGCGGCGTTCTTCTCG |
| JYI#79 | GAGAGCAAGTACGGACCACCCTG |
| JYI#80 | GGGTTTAAACGGGCCCTCTAGATTATCACTTGCCCAGGGACAGTGACAGG |
| JYI#81 | GAATTCCGCTCTAGATTATCAGGATTTTGGAGGATGGTCGCCACC |
| JY#28 | GGGGCCCAGCCGGCC |
| JY#29 | GGCCCCCGAGGCCCC |
| JY#30 | TACATTTTCAATTAAGATGCAGTTACTTCGCTGTTTTTCAATATTTTCTGTTATTGCTTCAGTTTTAGCA GGGGCCCAGCCGGCC |
| JY#31 | GATTTGCTCGCATATAGTTGTCAGTTCCTGAGAACCACCACCACCAGAACCACCACCACCAGAACCACCACCACC GGCCCCCGAGGCCCC |
| JY#33 | ATCCACTTTGCCTTTCTCTCCACAGGCGCGCACTCC |
| JY#34 | CAGCGGGTTTAAACGGGCCCTCTAGATTATCA |

**Table S2.** List of plasmids used in this study.

| Plasmids | Relevant Characteristics | Reference or source |
| --- | --- | --- |
| pMAZ-hICOS-His | *Human ICOS (21-129)-His* gene in pMAZ | This study |
| pMAZ-hICOS-GST | *Human ICOS (21-140)-GST* gene in pMAZ | This study |
| pMAZ-hICOS-Fc | *Human ICOS (21-140)-hFc* gene in pMAZ | This study |
| pMAZ-mICOS-Fc | *Mouse ICOS (21-141)-hFc* gene in pMAZ | This study |
| pMAZ-hPD-1-GST | *Human PD-1-GST* gene in pMAZ | ^[1]^ |
| pMAZ-hPD-1-Fc | *Human PD-1-hFc* gene in pMAZ | This study |
| pMAZ-ICOS-L-wild-type-His | *ICOS-L (Wild-type)-His* gene in pMAZ | This study |
| pMAZ-ICOS-L-A184-His | *ICOS-L (A184)-His* gene in pMAZ | This study |
| pMAZ-ICOS-L-Y8-His | *ICOS-L (Y8)-His* gene in pMAZ | This study |
| pMAZ-ICOS-L-H(N57H)-His | *ICOS-L (H)-His* gene in pMAZ | This study |
| pMAZ-ICOS-L-P(Q51P)-His | *ICOS-L (P)-His* gene in pMAZ | This study |
| pCTCON-Aga2-ICOS-L wild-type-FLAG | *ICOS-L (Wild-type)* gene in pCTCON-Aga2-FLAG | This study |
| pCTCON-Aga2-ICOS-L A184-FLAG | *ICOS-L (A184)* gene in pCTCON-Aga2-FLAG | This study |
| pCTCON-ICOS-L-wild-type-Aga2-FLAG | *ICOS-L (Wild-type)* gene in pCTCON-Aga2-FLAG | This study |
| pCTCON-ICOS-L-A184-Aga2-FLAG | *ICOS-L (A184)* gene in pCTCON-Aga2-FLAG | This study |
| pCTCON-ICOS-L-Y8-Aga2-FLAG | *ICOS-L (Y8)* gene in pCTCON-Aga2-FLAG | This study |
| pMAZ-ICOS-L-wild-type-Fc (LALAPG) | *ICOS-L (Wild-type)-IgG1 Fc (LALAPG)* gene in pMAZ | This study |
| pMAZ-ICOS-L-A184-Fc (LALAPG) | *ICOS-L (A184)-IgG1 Fc (LALAPG)* gene in pMAZ | This study |
| pMAZ-ICOS-L-Y8-Fc (LALAPG) | *ICOS-L (Y8)-IgG1 Fc (LALAPG)* gene in pMAZ | This study |
| pMAZ-pembrolizumab-IgL | *Pembrolizumab IgL* gene in pMAZ | This study |
| pMAZ-pembrolizumab-IgH | *Pembrolizumab IgH (S228P)* gene in pMAZ | This study |
| pMAZ-pembrolizumab-IgH (Fc-SH) | *Pembrolizumab IgH (Fc-SH)* gene in pMAZ | This study |
| pMAZ-ICOS-L-wild-type-Fc (Fc-SH) | *ICOS-L (Wild-type)-IgG4 Fc (Fc-SH)* gene in pMAZ | This study |
| pMAZ-ICOS-L-Y8-Fc (Fc-SH) | *ICOS-L (Y8)-IgG4 Fc (Fc-SH)* gene in pMAZ | This study |
| pMAZ-pembrolizumab-IgH (Fc-SH)-ICOS-L-Y8 | *Pembrolizumab IgH (Fc-SH)-ICOS-L Y8* gene in pMAZ | This study |
| pMAZ-pembrolizumab-IgL-ICOS-L-wild-type | *Pembrolizumab IgL-ICOS-L wild-type* gene in pMAZ | This study |
| pMAZ-pembrolizumab-IgL-ICOS-L-Y8 | *Pembrolizumab IgL-ICOS-L Y8* gene in pMAZ | This study |

**SI References**

1. Jo M, Ko S, Hwang B, Min SW, Ha JY, Lee JC, et al. Engineered human FcγRIIa fusion: A novel strategy to extend serum half‐life of therapeutic proteins. Biotechnol Bioeng. 2020;117(8):2351-61.
